# Ferroptosis-related gene signature-based subtype identification of triple-negative breast cancer to prioritize treatment strategies

**DOI:** 10.3389/fonc.2025.1541119

**Published:** 2025-05-20

**Authors:** Yongzhen Chen, Xiaoying Huang, Junqi Wang, Chao Hu, Yanan Zheng, Yumeng Wang, Shuqian Zheng, Guozhong Ji, Qiang You

**Affiliations:** ^1^ Department of General Practice, The Second Affiliated Hospital of Nanjing Medical University, Nanjing, China; ^2^ Laboratory of Frontiers Science Center for Precision Oncology, Faculty of Health Sciences, University of Macau, Macau, Macau SAR, China; ^3^ Department of Oncology and Hematology, Shenzhen Traditional Chinese Medicine Hospital , Shenzhen, China; ^4^ Department of Biotherapy, Department of Geriatrics, The Second Affiliated Hospital of Nanjing Medical University, Nanjing, China

**Keywords:** ferroptosis, triple-negative breast cancer, molecular subtype, immunotherapy, chemotherapy

## Abstract

**Purpose:**

Ferroptosis, an iron-dependent form of regulated cell death (RCD), has been proven to affect the response to antineoplastic therapies. However, little is known about the role of ferroptosis in chemotherapy and immune checkpoint inhibitor (ICI) therapy responses, as well as the molecular subtype identification of triple-negative breast cancer (TNBC).

**Methods:**

We performed unsupervised clustering to stratify patients with TNBC in the Fudan University Shanghai Cancer Center (FUSCC) TNBC cohort into distinct ferroptosis-related subtypes according to the expression of eight ferroptosis-related genes (FRGs): EMC2, FTH1, HMOX1, LPCAT3, NOX4, SOCS1, BAP1, and ISCU. We conducted Gene Ontology (GO) analysis and gene set variation analysis (GSVA) to characterize the immune phenotype and enriched pathways of the distinct subtypes of TNBC. We constructed the FerrScore model to identify the most promising candidate compounds and predict ICI therapy benefits for patients with TNBC.

**Results:**

We identified two distinct ferroptosis-related subtypes with different overall survival (OS). Patients in cluster 1 exhibited better OS, which had a phenotype of a “hot” tumor with abundant immune cell infiltration and higher expression of immune checkpoints compared to cluster 2. We screened everolimus as the most promising candidate drug for patients with high FerrScore referring to comprehensive factors including CMap score, experimental evidence, and clinical trial status. Further, we confirmed that FerrScore was a potentially powerful metric to predict anti-PD-L1, anti-PD-1, and anti-PD-1 + CTLA-4 ICI therapy benefits.

**Conclusions:**

Ferroptosis reprogrammed the tumor microenvironment (TME) and classified patients into distinct subgroups with significantly different OS. FerrScore was a potentially powerful metric to screen candidate compounds and predict ICI therapy benefits for patients with TNBC, which prioritized clinical treatment strategies.

## Background

Triple-negative breast cancer (TNBC), the most aggressive group of breast cancer with a high degree of intra-tumoral heterogeneity, accounts for approximately 15%–20% of all invasive breast tumors (1). TNBC is characterized by the absence of progesterone receptor (PR), estrogen receptor (ER), and human epidermal growth factor receptor 2 (HER-2) expression (2) with a median overall survival (OS) rarely exceeding 12 to 18 months in advanced cancers (3). Despite substantial aggressive therapeutic strategies that have been applied to clinical therapy, such as surgery, radiotherapy, chemotherapy, and immunotherapy, breast cancer has surpassed lung cancer as the leading cause of cancer-related death in women worldwide according to the Global Cancer Statistics 2020 (4). Chemotherapy remains a mainstay in the clinical treatment of unresectable TNBC in accordance with the 2022 version of the Chinese Society of Clinical Oncology (CSCO)-TNBC guidelines (5). Multiple studies have shown that immunotherapy increases the sensitivity of tumor cells to chemotherapy (6). In-depth studies and analysis about how chemotherapy and immunotherapy are used are therefore urgently needed for guiding the clinical therapy of TNBC.

Molecular subtype classification is essential for understanding cancer biology and guiding precision therapy (7, 8), particularly in TNBC (9). First, Lehmann identified six TNBC subtypes with unique gene expression profiles and ontologies, including two basal-like (BL1 and BL2), mesenchymal (M), mesenchymal stem-like (MSL), luminal androgen receptor (LAR), and immunomodulatory (IM) subtypes, by cluster analysis to guide therapeutic decision (10). Subsequently, Lehmann refined TNBC molecular subtypes from six into four tumor-specific subtypes (LAR, BL1, BL2, and M) (11). Afterward, Professor Powel stratified TNBC into four distinct subtypes—basal-like immune-activated (BLIA), basal-like immunosuppressed (BLIS), LAR, and mesenchymal-like (MES)—by RNA and DNA profiling analyses (12, 13). However, these findings do not actually rewrite the clinical practice guidelines and improve the outcome of patients with TNBC. Immediately after, Professor Zhimin Shao classified TNBC into four subtypes—BLIS, MES, LAR, and IM subtypes—according to genomic and transcriptomic profiles and proposed Fudan University Shanghai Cancer Center (FUSCC) subtype-based precision therapeutic strategies (14). Recently, Shao’s group identified three distinct metabolomic subgroups to further advance the understanding and precision therapy of TNBC (15). Although a large number of molecular subtyping approaches were proposed to serve as references for precision therapy, there still remained some limitations and bottlenecks due to the high intra-tumoral heterogeneity of TNBC. Therefore, a new classification system study is still an exciting focus for TNBC in the future.

Ferroptosis, an iron-dependent form of regulated cell death (RCD), has been proven to affect the response to radiotherapy, chemotherapy, and immunotherapy of tumors (16). Previous studies have suggested that breast cancer cells were more sensitive to ferroptosis resulting from CD44-dependent iron endocytosis, which promoted the activity of iron-dependent demethylases to upregulate epithelial–mesenchymal transition (EMT) pathway-related genes (17). Especially, the human TNBC cell line (MDA-MB-157) is more sensitive to RSL3 (ferroptosis activator)-triggered ferroptosis for the high expression of ACSL4 (18). Published studies have uncovered that TNBC is enriched in ferroptosis-related gene signature and co-inhibition of BET and that proteasome induced ferroptosis in all major TNBC subtypes (BL1/2 and M/MSL), while targeting BET and CXCR2 induced tumor cell apoptosis in mesenchymal TNBC subtype (19). Recent research has revealed the potential roles of ferroptosis in TNBC (20–22). Ferroptotic tumor cell-derived damage-associated molecular patterns (DAMPs) triggered inflammation-related immunosuppression and immune landscape reshaping in the tumor microenvironment (TME), thus contributing to tumor growth (23). In addition, the latest research indicated that ferroptotic cancer cells recruited T cells, NK cells, and macrophages, thereby turning “cold” tumors into “hot” phenotypes by activating antitumor immune responses (24). The above illustrates ferroptosis as a promising direction for precision therapy of TNBC. Ferroptosis-related signatures have been applied to the molecular subtyping of several cancer types, for instance, pancreatic ductal adenocarcinoma, lung adenocarcinoma, and hepatocellular carcinoma (25–27). Further interrogating the role of ferroptosis in the molecular subtyping of TNBC, as well as in TME remodeling, is therefore of paramount importance.

In the present study, we first identified eight ferroptosis-related genes (FRGs) and explored the gene expression heterogeneity in pan-cancers. We then focused on the FRG-based molecular subtype identification of TNBC according to the mRNA expression of the eight FRGs and characterized the immune landscape within distinct subtypes. Further, we constructed a ferroptosis-related scoring model by the product of mRNA expression and correlation coefficient of the eight FRGs and defined the ferroptosis-related gene signature as the FerrScore. We identified six Cancer Therapeutics Response Portal (CTRP)-derived and six Profiling Relative Inhibition Simultaneously in Mixtures (PRISM)-derived drugs based on FerrScore and further confirmed everolimus as the most promising candidate drug for TNBC patients with high FerrScore. Meanwhile, we applied FerrScore to immunotherapy cohorts to evaluate the diagnostic value of FerrScore in immune checkpoint inhibitor (ICI) therapy benefit prediction and found that FerrScore was a promising biomarker to predict anti-PD-1, anti-PD-L1, and anti-PD-1 + CTLA-4 therapy benefits. Taken together, the FRG signature is a potentially powerful metric to stratify patients with TNBC, and FerrScore is a reliable basis to screen candidate compounds and predict ICI therapy benefits, which provides new perspectives for clinical treatment.

## Materials and methods

### Data acquisition and filtration

The transcriptomic profiles and clinical information of patients with TNBC were obtained from the National Omics Data Encyclopedia (NODE) (https://www.biosino.org/node/) (FUSCC, Project ID: OEP000155) conducted by Zhimin Shao from Fudan University Shanghai Cancer Center, and finally, 358 tumor samples and 88 normal samples were enrolled into further analysis after excluding patients without matched information (14). The GSE76124 and GSE21653 cohorts were used as the validation datasets (12).

### Genetic variations and expression profiles of FRGs

A total of 125 FRGs were first identified from the FerrDb (http://www.zhounan.org/ferrdb/legacy), and 841 survival-related genes were screened from the FUSCC dataset by the Kaplan–Meier survival analysis. Finally, nine hub genes were chosen after an intersection. Ultimately, eight FRGs were applied for the unsupervised clustering analysis, while ALOX15B was excluded with no expression difference between normal tissues and tumors. The expression profiles of the eight FRGs were visualized at the single-cell level in GSE118389, and they were further validated in multiple single-cell RNA-seq datasets from Tumor Immune Single-cell Hub (TISCH) (http://tisch.comp-genomics.org/home/). In addition, genetic variations of the eight FRGs were elaborated using the “maftools” R package in the Cancer Genome Atlas Program (TCGA) TNBC cohort. The dysregulation and methylation profiles of the eight FRGs were also illustrated, as well as Pearson’s correlation between FRG expression and copy number variation (CNV), and FRG expression and promoter methylation in pan-cancer.

### FRG-based unsupervised clustering

Unsupervised clustering analysis was performed according to the mRNA expression of the eight FRGs using the “ConsensusClusterPlus” R package to identify molecular subtypes, and principal component analysis (PCA) was applied to demonstrate the distribution of FRG-related subtypes. The Kaplan–Meier analysis was used to compare the survival probability between distinct groups. The reliability and stability of our FRG-related subtype identification strategy in the FUSCC TNBC cohort were then verified by the Non-negative Matrix Factorization (NMF) algorithm using the “CancerSubtypes” R package, as well as in the GSE76124 and GSE21653 cohorts using the “ConsensusClusterPlus” R package.

### Gene Ontology analysis and gene set variation analysis between distinct subtypes of TNBC

We screened the differentially expressed genes (DEGs) between different ferroptosis subtypes using the “limma” R package with a fold-change of 1.5 and an adjusted p-value of <0.05. We then applied the “ClusterProfiler” R package to perform Gene Ontology (GO) analysis to explore the involved biological processes of the ferroptosis-related subtypes of TNBC. In order to further characterize the immune characteristics within the distinct ferroptosis subtypes, we analyzed the immune-related pathways by gene set variation analysis (GSVA) according to the Molecular Signatures Database (MSigDB-v5.2) (https://www.gsea-msigdb.org/gsea/index.jsp).

### Tumor immune landscape characterization within distinct subtypes of TNBC

We compared the immune score, stromal score, estimate score, tumor purity, Tumor Immune Dysfunction and Exclusion (TIDE) score, exclusion score, and dysfunction score using the “ESTIMATE” R package and TIDE (http://tide.dfci.harvard.edu/), as well as tumor inflammation signature (TIS) score according to the mRNA expression of the 18 genes (28). We estimated immune cell infiltration between different groups using the Cibersort, XCELL, ssGSEA, TIMER, and MCP counter algorithms.

### Construction of the FerrScore scoring model

To further explore the potential diagnostic value of ferroptosis signatures to prioritize treatment strategies of TNBC, the penalized Cox regression model based on the least absolute shrinkage and selection operator (LASSO) penalties was used to calculate the correlative coefficients of the eight FRGs and defined the ferroptosis-related gene signature as the FerrScore. The FerrScore formula was established as follows:

FerrScore = ∑ (correlative coefficient × gene’s expression).

Patients with TNBC were divided into high and low FerrScore groups according to the optimal cut-off value using the “Survminer” R package, and the OS was compared between groups. The “survivalROC” R package was used to plot the receiver operating characteristic (ROC) curve and estimate the predictive accuracy of the FerrScore model.

### FerrScore-based drug sensitivity screening

To further screen the promising agents for patients with high FerrScore, the FerrScore construction model was applied to the PRISM (https://www.theprismlab.org/) and CTRP2.0 (https://portals.broadinstitute.org/ctrp/) databases. The expression profile of the cancer cell lines (CCLs) was available on the Broad Institute-Cancer Cell Line Encyclopedia (CCLE) portal (https://portals.broadinstitute.org/ccle/). The area under the dose–response curve (AUC) values were calculated to estimate the drug sensitivity, and lower AUC values indicated higher sensitivity to chemotherapy. Spearman’s correlation analysis was performed to explore the association between FerrScore and AUC values to identify the candidate drugs for patients with high FerrScore. Further, the CMap score of drugs was calculated by submitting the 300 DEGs between normal tissues and tumors with the most significant fold changes (150 up- and 150 downregulated genes) to the CMap website (https://clue.io/query). Clinical trial status, experimental evidence, and the mRNA expression of drug targets were obtained from the National Center for Biotechnology Information (NCBI) (https://pubmed.ncbi.nlm.nih.gov/) and DrugBank (https://go.drugbank.com/).

### FerrScore-based ICI therapy benefit prediction

To further evaluate the diagnostic value of FerrScore in ICI therapy benefit prediction, a urothelial cancer cohort was applied using the “IMvigor210CoreBiologies” R package to explore the association between FerrScore and anti-PD-L1 immunotherapy response. Further, the GSE78220 and PRJEB23709 cohorts were applied to study the potential role of FerrScore in anti-PD-1 and anti-PD-1 + CTLA-4 ICI therapy, and adoptive T-cell therapy response in the GSE100797 cohort, respectively.

### Histological analysis

Human TNBC samples were obtained from the Second Affiliated Hospital of Nanjing Medical University (Nanjing, China). Tissues were sectioned into 5-μm-thick paraffin sections and further subjected to hematoxylin and eosin (H&E) and immunohistochemical (IHC) staining with anti-SOCS1 Rabbit pAb (Cat# GB11372, ServiceBio, Hubei, China), anti-NOX4 Rabbit pAb (Cat# GB11347, ServiceBio), anti-PD-1 Rabbit pAb (Cat# GB11338, ServiceBio), and anti-CD3 Rabbit pAb (Cat# GB11014, ServiceBio).

### Four-color immunofluorescence

Formalin-fixed paraffin-embedded sections were stained with anti-SOCS1 Rabbit pAb (Cat# GB11372, ServiceBio), anti-NOX4 Rabbit pAb (Cat# GB11347, ServiceBio), anti-PD-1 Rabbit pAb (Cat# GB11338, ServiceBio), and anti-CD3 Rabbit pAb (Cat# GB11014, ServiceBio) overnight at 4°C and then incubated with secondary antibodies for 1 hour at room temperature, while the cell nucleus was counterstained with DAPI (Cat# GDP1024, ServiceBio).

### *In vitro* cell viability assay

MDA-MB-231 TNBC cells were transfected with pshRNA-SOCS1 plasmids (targeting 5′-CACGCACTTCCGCACATTC-3′) (29) or control plasmids using Lipofectamine™ 3000 reagent. The knockdown efficiency was evaluated by Western blotting analysis using an antibody against human SOCS1 (Cat# 55313, Cell Signaling Technology, Inc., Danvers, MA, USA). Then, the cells (4.0 × 10^3^/well) were seeded into 96-well plates, incubated overnight, and treated with RSL3 (0.5 μM) (Cat# HY-100218A, MedChemExpress, Monmouth Junction, NJ, USA) for 72 hours. Cell viability was assessed using the Cell Counting Kit-8 (CCK-8 Kit) (Vazyme, Nanjing, China) according to the manufacturer’s instructions.

### Statistical analysis

The R version 4.0.4 software was used for the RNA-seq data analysis and visualization. Wilcoxon test and Student’s t-test were performed to compare the differences between the two groups, while the Kruskal–Wallis test and one-way ANOVA were used to compare the differences between three or more groups. Spearman’s and Pearson’s correlation analyses were chosen to explore whether there was a positive or negative relationship. The Kaplan–Meier survival analysis was applied to compare the survival probability across groups. For comparisons among groups, p < 0.05 indicates significant differences.

## Results

### Identification, genetic variations, and expression profiles of the FRGs

**Figure 1:**
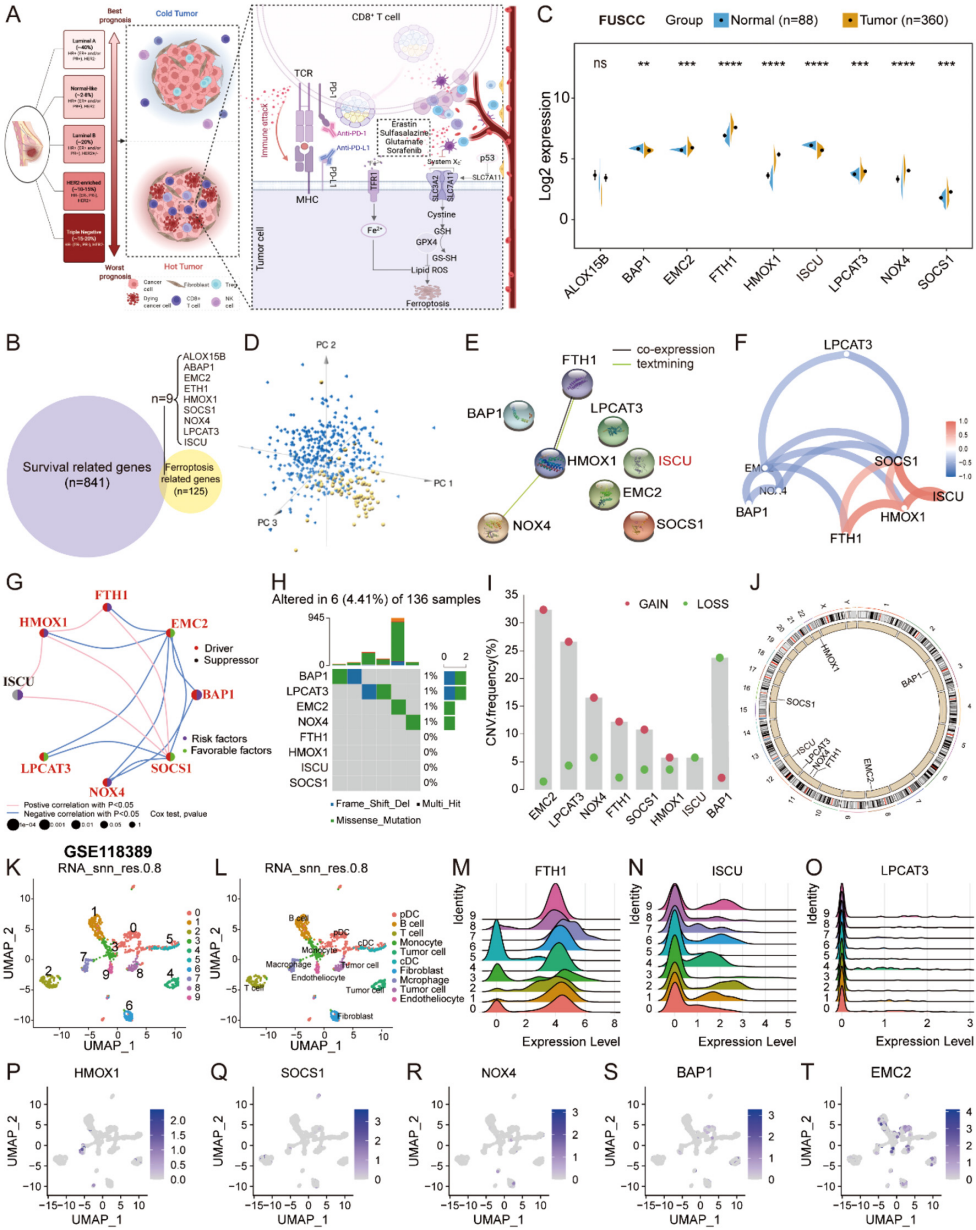
Expression profiles and genetic variations of the FRGs. **(A)** Overview of the cytotoxic T cell-driven immunity promotes ferroptosis in cancer cells to reshape the TME in TNBC drawn by BioRender (https://biorender.com/). **(B)** The Venn diagram was plotted to show the identification of crucial FRGs in FUSCC TNBC. **(C)** The expression profiles of the FRGs between normal tissues and tumors displayed by split violin plot; Student’s t-test, ** P < 0.01, *** P < 0.001, **** P < 0.0001, NS, no significance. **(D)** PCA to distinguish tumors from normal tissues according to the expression of the eight FRGs in FUSCC TNBC cohort. **(E–G)** PPI network and the correlation of the eight FRGs in FUSCC TNBC cohort. **(H–J)** The somatic mutation, CNV frequency, and chromosomal location of CNV of the eight FRGs in TCGA TNBC. **(K–L)** Uniform Manifold Approximation and Projection (UMAP) plot of total cells from patients with TNBC in GSE118389, with each cell color coded for cluster and cell type. **(M–T)** The expression profiles of eight FRGs in TNBC at single-cell level. FRGs, ferroptosis-related genes; TME, tumor microenvironment; TNBC, triple-negative breast cancer; FUSCC, Fudan University Shanghai Cancer Center; PCA, principal component analysis; PPI, protein–protein interaction; CNV, copy number variation.

Cytotoxic T cell-driven immunity promotes ferroptosis in cancer cells; for instance, anti-PD-L1 antibody induces lipid peroxidation-dependent ferroptosis in tumor cells (**Figure 1A**), which made ferroptosis-related anticancer therapy a promising target (30). Therefore, we designed the present study to explore the role of ferroptosis in the molecular subtype identification of TNBC and constructed a FerrScore model to screen promising drugs and predict response to immunotherapy (**Supplementary Figure 1**). We first identified 125 FRGs from the FerrDb and intersected them with 841 survival-related genes screened using the Kaplan–Meier survival analysis (**Figure 1B**, **Supplementary Figures 2A–J**, **Supplementary Table 1**). In FUSCC TNBC cohort, EMC2, FTH1, HMOX1, LPCAT3, NOX4, and SOCS1 were upregulated in tumors, while BAP1 and ISCU were downregulated (**Figure 1C**). Eight hub genes were identified, while ALOX15B was excluded for no difference between normal tissues and tumors, and the expression of the eight FRGs distinguished tumors from the normal tissues by PCA (**Figure 1D**). In addition, the expression of the eight FRGs varies in different grades and T and N stages of TNBC (**Supplementary Figure 3**). The length of the coding sequence (CDS) and chromosomal location of the eight FRGs were obtained from the NCBI (**Supplementary Table 2**). In pan-cancer analysis, ISCU was obviously downregulated in breast invasive carcinoma, and EMC2 suffered the highest frequency of CNV (**Supplementary Figures 4A,B**). NOX4 showed the highest frequency of methylation, while the expression of SOCS1 positively correlated with the promoter methylation across 20 types of cancer in TCGA (**Supplementary Figures 4C, D**).

Protein–protein interaction (PPI) network showed that HMOX1 interacted with FTH1 and NOX4, and the correlation of the eight FRGs is exhibited in the network plot (**Figures 1E–G**). Somatic mutation analysis of the eight FRGs showed that 6 (4.41%) of 136 samples suffered genetic variations, and EMC2 underwent dramatically high frequency (32.37%) of copy number gain in TCGA cohort (**Figures 1H–J**, **Supplementary Table 2**). In the GSE118389 scRNA-seq dataset, SOCS1 was primarily expressed in T cells, and HMOX1 was principally expressed in macrophages at the single-cell level (**Figures 1K–T**), as well as validated in another four datasets in TISCH (**Supplementary Figures 5A–H**). At the protein level, FTH1, ISCU, and SOCS1 were medium stained in breast cancer tissues on the Human Protein Atlas (HPA) portal (**Supplementary Figure 5I**). The results strongly indicated that dysregulation and imbalance of the FRGs contributed to the progression and malignancy of TNBC.

### Ferroptosis-related subtype identification and pathway enrichment analysis

**Figure 2:**
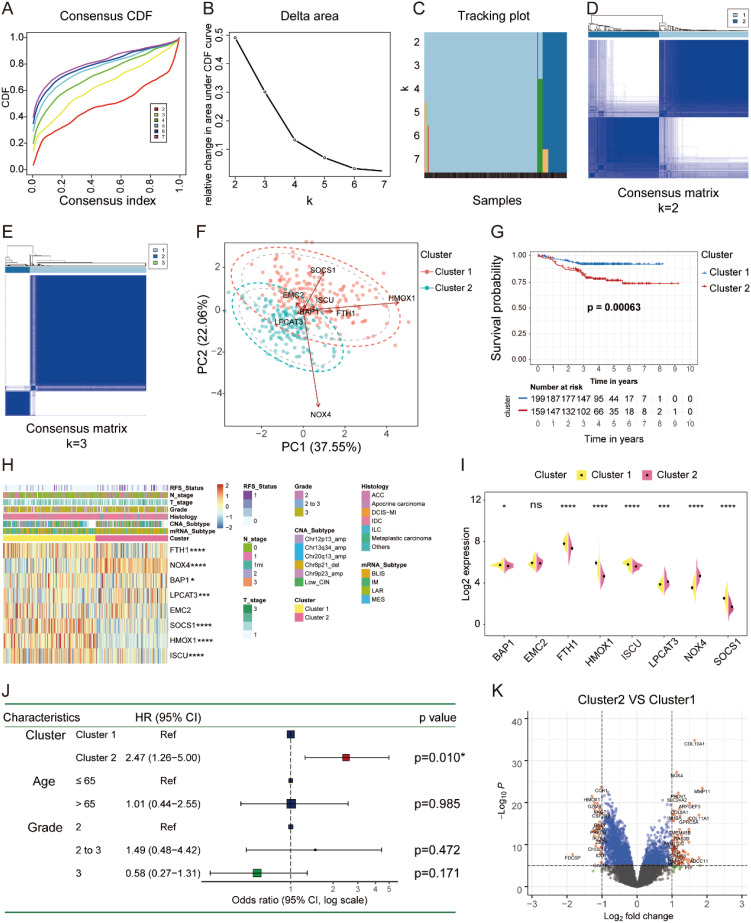
Ferroptosis subtype identification in FUSCC TNBC cohort. **(A–E)** The consensus CDF, delta area, tracking plot, and consensus matrix (K = 2, 3) plotted by “ConsensusClusterPlus” R package to identify distinct clusters in patients with TNBC. **(F)** PCA revealed that the expression of the eight FRGs represented by distinct ferroptosis subtypes. **(G)** Kaplan–Meier curve showed the significant difference in OS between the two molecular subtypes of TNBC. **(H, I)** The heatmap and split violin plot illustrated the variations of the eight FRGs among different subtypes of TNBC. **(J)** Univariate Cox regression between the two ferroptosis subtypes on OS in TNBC by forest plot. **(K)** Volcano plot showed the DEGs between different subtypes of TNBC (fold change = 1.5). FUSCC, Fudan University Shanghai Cancer Center; TNBC, triple-negative breast cancer; PCA, principal component analysis; FRGs, ferroptosis-related genes; OS, overall survival; DEGs, differentially expressed genes. * P < 0.05, *** P < 0.001, **** P < 0.0001. NS, no significance.

Consensus clustering was performed to identify ferroptosis-related molecular subtypes according to the mRNA expression of the eight FRGs in the FUSCC TNBC cohort. Patients were divided into two distinct groups (K = 2) with high and low intergroup correlations (**Figures 2A–E**). PCA exhibited that the distinct subgroups can be distinguished by the expression of the eight FRGs (**Figure 2F**). Patients in cluster 1 showed better OS than those in cluster 2 by the Kaplan–Meier analysis (**Figure 2G**). Tumors in cluster 2 were indeed mainly composed of IM and BLIS mRNA subtypes with younger age distribution (**Supplementary Figures 6A–C**, **Supplementary Table 3**). Research from the MD Anderson Cancer Center suggested that the BLIS mRNA subtype had the worst outcome (13). The expression of LPCAT3 and NOX4 was higher in cluster 2, and EMC2 showed no difference, while others exhibited an opposite profile (**Figures 2H, I**). Univariate Cox regression analysis showed that cluster 2 was a risk factor [Hazard Ratio (HR) = 2.47] with worse OS (**Figure 2J**). As validated by the NMF Clustering algorithm, patients in the FUSCC TNBC cohort were also divided into two distinct subgroups with significantly different OS probabilities (**Supplementary Figures 7A–C**). Similarly, patients with TNBC were clearly divided into two groups with different OS using unsupervised clustering analysis in the GSE76124 and GSE21653 datasets, and PCA showed the distribution of FRG-related subtypes (**Supplementary Figures 7D–K**).

**Figure 3:**
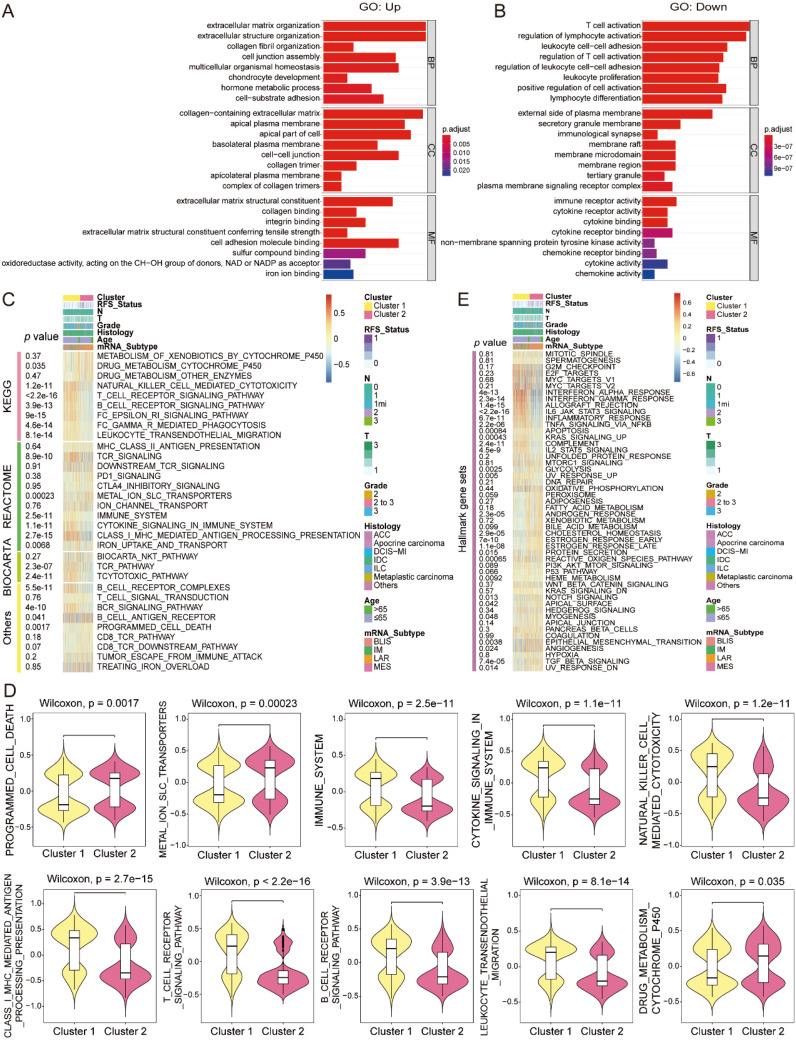
GO and GSVA of the two distinct subtypes. **(A, B)** GO analysis of up- and downregulated genes between the two subtypes (cluster 2 vs. cluster 1). **(C–E)** GSVA between two distinct subtypes by MSigDB-v5.2 of C2 curated gene sets and H hallmark gene sets. GO, Gene Ontology; GSVA, gene set variation analysis.

To further explore the involved GO processes and enriched pathways that participated in the progression of distinct subgroups, we identified the DEGs by setting the fold change of 1.5 as illustrated in the volcano plot (**Figure 2K**). GO analysis of the upregulated DEGs showed that iron ion binding molecular function (MF) was significantly enriched in cluster 2, while the downregulated DEGs were predominately enriched in T-cell activation and leukocyte proliferation biological processes (BPs) (**Figures 3A, B**). Altogether, tumors in cluster 2 with worse OS exhibited more iron ion overloading and less T-cell activation, which strongly suggested that tumors in cluster 2 were more sensitive to ferroptosis. Afterward, we performed GSVA to further study the impact of tumor cell ferroptosis on TME reprogramming and found that tumors in cluster 2 exhibited higher enrichment scores of programmed cell death, metal ion SLC transporters, drug metabolism cytochrome p450, and EMT pathways (**Supplementary Figure 8**). A previous study confirmed that tumor cells with a high mesenchymal state were more sensitive to ferroptosis as enhanced iron endocytosis during EMT (17, 31). Additionally, tumors in cluster 2 showed lower enrichment scores of immune-related pathways, such as natural killer cell-mediated cytotoxicity, leukocyte transendothelial migration, and T-cell and B-cell receptor signaling pathways (**Figures 3D, E**, **Supplementary Table 4**). Altogether, tumors in cluster 2 were a group of cancer types that were more sensitive to ferroptosis with an immunosuppressive phenotype and worse OS compared to that of cluster 1.

### Immune landscape characterization within ferroptosis-related subtypes

**Figure 4:**
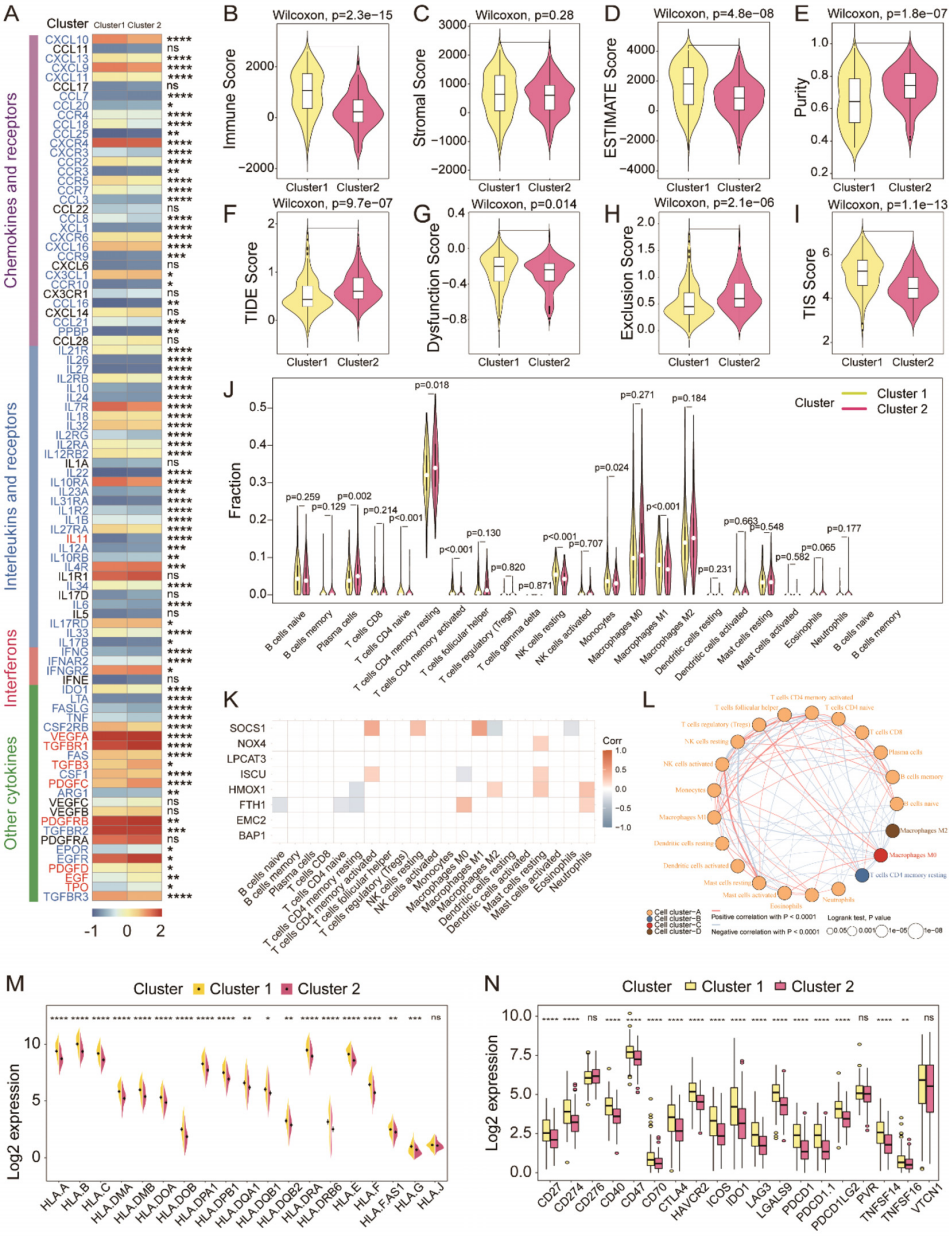
Variations of immune-related regulators and TME landscapes within distinct ferroptosis phenotypes. **(A)** The heatmap describes the variations in mRNA expression of chemokines, interleukins, interferons, and other cytokines in ferroptosis phenotypes; Student’s t-test, *p < 0.05, ** P < 0.01, *** P < 0.001, **** P < 0.0001. **(B–I)** The immune, stromal, ESTIMATE, TIDE, dysfunction, exclusion, and TIS scores and tumor purity among the two subtypes; Wilcoxon test. **(J)** Infiltration of 24 populations of immune cells within the two clusters by Cibersort; Student’s t-test. **(K)** Spearman’s correlation between the expression of the eight FRGs and immune cell infiltration in TNBC. **(L)** Interaction network of infiltrated immune cells by Spearman’s correlation analysis according to the expression of the eight FRGs, and the line connecting two cell types indicates an interaction between them. **(M, N)** Variations of MHC complexes and immune checkpoint expression at mRNA level among the ferroptosis subtypes; Student’s t-test. TME, tumor microenvironment; FRGs, ferroptosis-related genes; TNBC, triple-negative breast cancer; NS, no significance.

Ferroptotic tumor cells release DAMPs to affect the infiltration, differentiation, and function of immune cells in the TME to influence tumor growth (23). We therefore performed comprehensive analysis to amply illustrate the immune signature of the two ferroptosis-related subtypes, as well as the role of ferroptosis in TME remodeling. The expression of chemokines, interleukins, interferons, and receptors were markedly different within the two distinct subgroups (**Figure 4A**). Tumors in cluster 2 possessed lower immune, stromal, ESTIMATE, dysfunction, and TIS scores, as well as higher tumor purity, TIDE score, and exclusion score (**Figures 4B–I**). Further, we applied Cibersort to infer the immune infiltration in tumors and found that there were less abundant NK cells resting and T cells CD4 memory activated in cluster 2 (**Figure 4J**), which may result from the low expression of SOCS1 that was positively correlated with NK cells resting numbers by Spearman’s correlation analysis (**Figure 4K**). In addition, the network plot illustrated the complex communications among immune cells in the TME of TNBC (**Figure 4L**). Finally, tumors in cluster 2 expressed less MHC complex and higher immune checkpoints (**Figures 4M, N**). In summary, tumors in cluster 2 were more likely to have a cold tumor phenotype with less immune infiltration and high expression of immune checkpoints to build an immunosuppressive TME.

### Construction of the FerrScore model in TNBC

**Figure 5:**
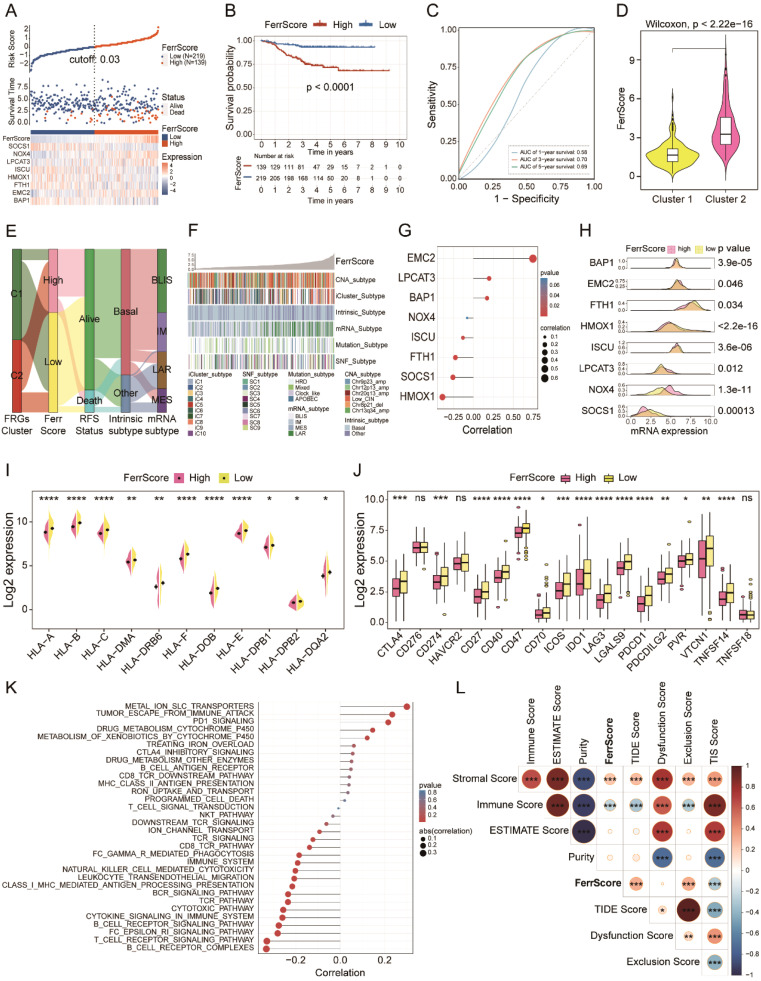
FRG-based FerrScore model construction in TNBC. **(A)** High and low FerrScore groups classification using “ggrisk” R package by the cut-off value of 0.03. **(B)** The Kaplan–Meier curve uncovered worse OS of patients with high FerrScore. **(C)** The ROC curve shows the specificity and sensitivity of FerrScore in predicting the 1-, 3-, and 5-year survival of patients with TNBC. **(D)** Variations of FerrScore between the two distinct clusters; Wilcoxon test. **(E)** The Sankey diagram displays the correlation between clusters, FerrScore, RFS status, intrinsic subtype, and mRNA subtype by “ggalluvial” R package. **(F)** The heatmap shows the correlation between FerrScore and Copy Number Aberration (CNA) subtype, iCluster subtype, intrinsic subtype, mRNA subtype, mutation subtype, and SNF subtype in patients with TNBC. **(G)** Pearson’s correlation between the expression of eight FRGs and FerrScore. **(H)** The mRNA expression of the eight FRGs among high and low FerrScore groups; Student’s t-test. **(I, J)** The expression of MHC complexes and immune checkpoints between the high and low FerrScore groups; Student’s t-test, *p < 0.05, ** P < 0.01, *** P < 0.001, **** P < 0.0001. **(K, L)** Pearson’s correlation analysis between FerrScore and immune-related signaling pathways and scores. FRG, ferroptosis-related gene; TNBC, triple-negative breast cancer; OS, overall survival; ROC, receiver operating characteristic; RFS, relapse-free survival; NS, no significance.

We constructed a FerrScore model based on eight FRGs to further study how ferroptosis affects the tumor phenotype and TME remodeling, and we evaluated the accuracy of FRG signature in predicting the response to chemotherapy and immunotherapy of TNBC. We identified high and low FerrScore groups with remarkably different OS by a cut-off value of 0.03, and the AUC of 3-year survival was 0.70 of the FerrScore model (**Figures 5A–C**, **Supplementary Table 5**). The age distribution, mRNA type, intrinsic subtype, paclitaxel treatment status, tumor grade, and relapse-free survival (RFS) status of patients among the two groups were different (**Supplementary Table 6**). Among the two distinct subtypes of TNBC, the FerrScore of cluster 2 was significantly higher than that of cluster 1, and patients in cluster 2 were finally incorporated into the high FerrScore group with worse OS (**Figures 5D, E**). We found that the high FerrScore group had more LAR mRNA subtype and less basal intrinsic subtype (**Figure 5F**). Among the eight FRGs, the expression of EMC2 was positively correlated with the FerrScore of tumors, while HMOX1 showed an obvious negative correlation (**Figure 5G**). The expression of LPCAT3, EMC2, and NOX4 was higher in the high FerrScore group than the lower one, while another five FRGs were downregulated in the high FerrScore group (**Figure 5H**).

We explored the immune signature within the high and low FerrScore groups to study the impact of FerrScore on TME reprogramming and finally noticed that FerrScore was negatively correlated with the expression of MHC complex molecules and immune checkpoints (**Figures 5I, J**). Meanwhile, we found that FerrScore positively correlated with metal ion SLC transporters, tumor escape from immune attack, and PD-1 signaling pathways, as well as negatively correlated with T-cell and B-cell receptor signaling pathways (**Figure 5K**). In addition, we also observed a positive association between FerrScore and stromal score, TIDE score, and exclusion score, along with a negative correlation between FerrScore and TIS score and immune score (**Figure 5L**). We inferred the immune signatures between the two groups using the Cibersort, XCELL, ssGSEA, TIMER, and MCP counter algorithms and found that tumors with low FerrScore had higher abundance of immune cells in the TME (**Supplementary Figure 9**). Altogether, FerrScore was an effective reference indicator to determine the immune phenotype of TNBC, which laid a solid foundation for its role in promising candidate drug screening and ICI therapy response prediction.

### FerrScore-based candidate drug screening to guide chemotherapy

Large numbers of preclinical evidence uncovered that the induction of ferroptosis in tumors contributed to preventing acquired chemotherapy resistance to several cancer therapies, and ferroptosis inducers have been proven to work synergistically with traditional drugs to suppress tumor growth in mouse models with head and neck cancer (32). We first applied the FerrScore scoring model to human breast cancer cell lines in CCLE and also identified two clusters (**Supplementary Figure 10A**). Tumors in cluster 2 were more sensitive to NUTLIN-3A(−), AFATINIB, and AZD8055 with lower AUC values, while tumors in cluster 1 were more sensitive to KU-55933, MASITINIB, PAC-1, AZD7762, BI-2536, and PELITNIB (**Supplementary Figures 10B–J**).

**Figure 6:**
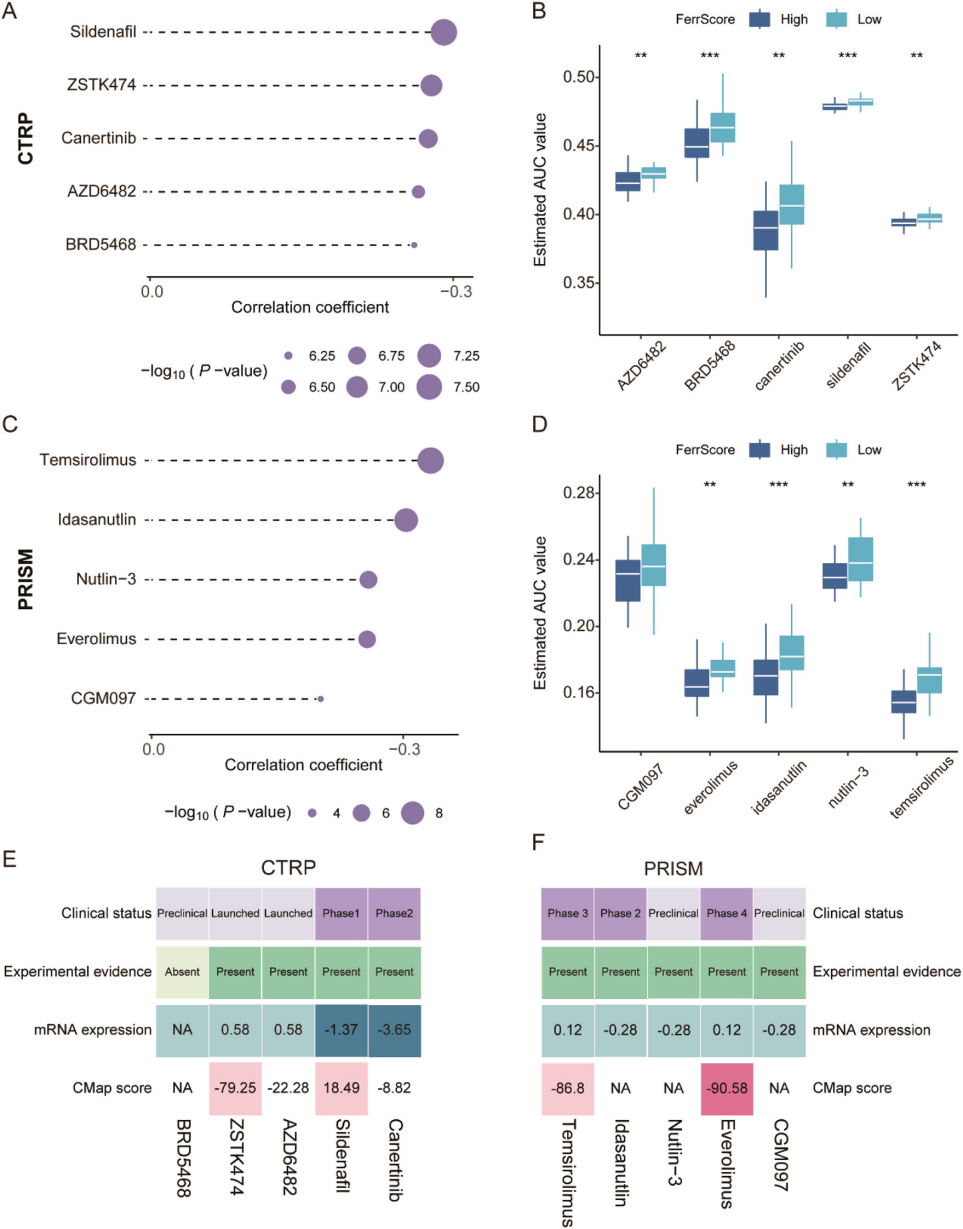
Promising candidate drug identification for TNBC patients with high FerrScore. **(A, B)** Spearman’s correlation analysis and drug response comparison of five CTRP-derived agents. **(C, D)** Spearman’s correlation analysis and drug response analysis of five PRISM-derived agents. **(E, F)** The clinical status, experimental evidence, mRNA expression of target genes, and CMap score of the five CTRP-derived and five PRISM-derived drugs documented by the heatmap. TNBC, triple-negative breast cancer; CTRP, Cancer Therapeutics Response Portal; PRISM, Profiling Relative Inhibition Simultaneously in Mixtures. ** P < 0.01, *** P < 0.001.

To further study the potential role of FerrScore in response to chemotherapy, we screened candidate compounds according to the FerrScore to guide the chemotherapy of patients with TNBC. First, we applied the FerrScore model to the CTRP and PRISM datasets and identified five CTRP-derived (BRD5468, ZSTK474, AZD6482, sildenafil, and canertinib) and five PRISM-derived (temsirolimus, idasanutlin, everolimus, CGM097, and Nutlin-3) drugs by Pearson’s correlation analysis for tumors with high FerrScore (**Figures 6A–D**, **Supplementary Table 7**). Second, we calculated the fold changes of the candidate agents’ target genes, while a higher fold change indicated more potential for candidate drugs. Third, to identify the most promising agents for high FerrScore tumors, we then calculated the CMap score by submitting the 300 DEGs with the most significant fold changes to the CMap website, and everolimus achieved the best grades with a CMap score of −90.58 (**Figures 6E, F**). Finally, we searched the published experimental evidence and clinical trial status of candidate drugs and found that everolimus had been acknowledged as a therapeutic agent for advanced breast cancer (**Supplementary Table 7**), which further confirmed that everolimus was the most promising compound for patients with higher FerrScore.

### FerrScore-based ICI therapy prediction to optimize immunotherapy

ICI therapy revolutionized the clinical treatment of patients with cancer. Anti-PD-L1 antibodies have been proven to act synergistically with ferroptosis activators (such as erastin) to suppress tumor cell growth (30). DAMPs released from ferroptotic tumor cells altered the TME status and ICI therapy response. Therefore, we applied the FerrScore scoring model to anti-PD-L1, anti-PD-1, and anti-PD-1 + CTLA-4 ICI immunotherapy and adoptive T-cell therapy cohorts to predict immunotherapy benefits.

**Figure 7:**
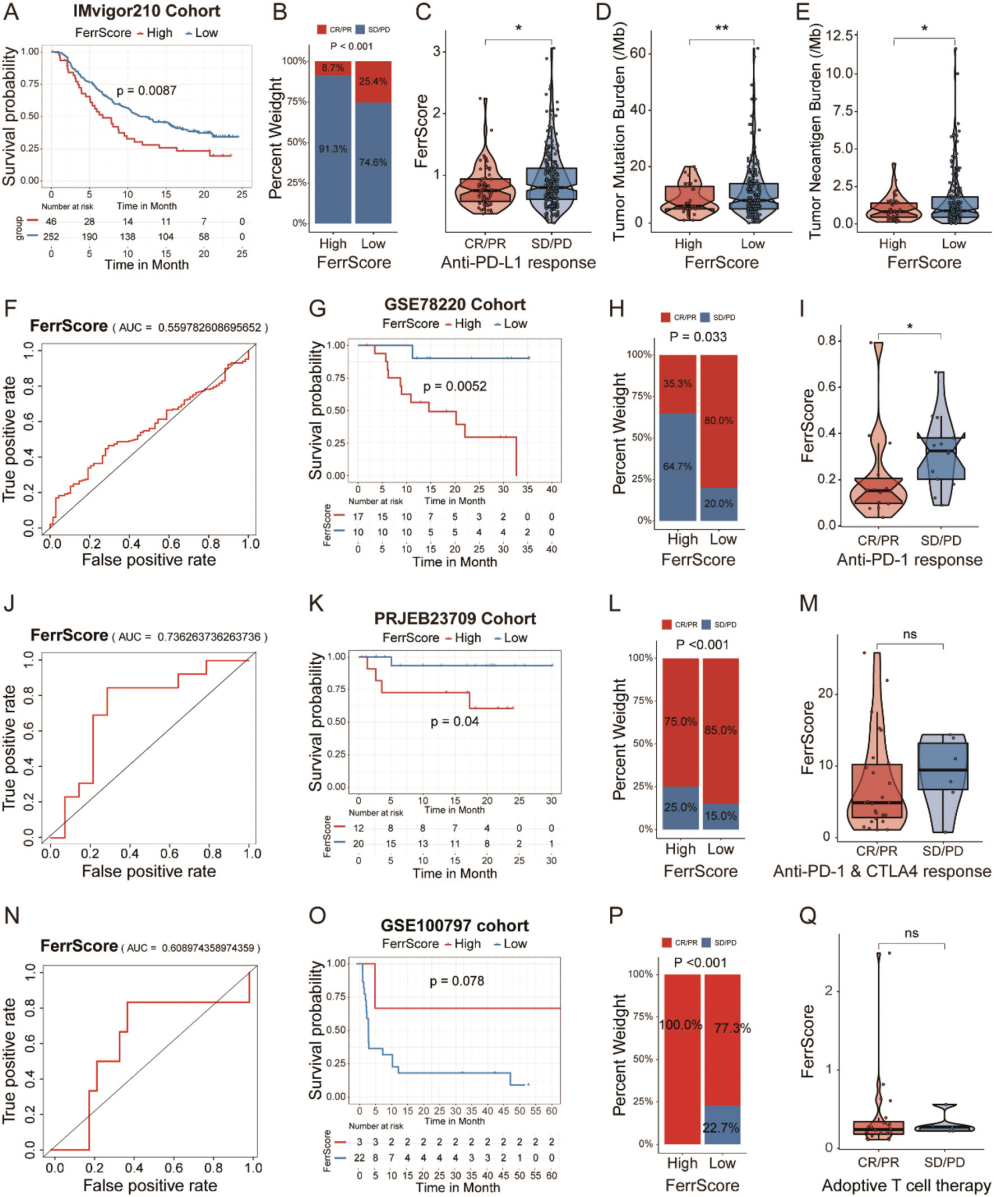
Prognostic value of the FerrScore in predicting ICI therapy benefits. **(A)** Kaplan–Meier survival analysis between patients with high and low FerrScore in IMvigor210 cohort with anti-PD-L1 therapy in urothelial carcinoma. **(B)** Variations of anti-PD-L1 responsiveness within high and low FerrScore groups; Student’s t-test. CR, complete response; PR, partial response; SD, stable disease; PD, progressive disease. **(C)** FerrScore of patients in different anti-PD-L1 response groups; Wilcoxon test, *p < 0.05. **(D, E)** TMB and TNB frequency between high and low FerrScore groups; Wilcoxon test, *p < 0.05, ** P < 0.01. **(F)** The ROC curve of FerrScore in predicting responsiveness of patients with anti-PD-L1 therapy. **(G)** Kaplan–Meier survival curve among high and low FerrScore groups in GSE78220 cohort with anti-PD-1 therapy in melanoma. **(H)** Difference in anti-PD-1 response between high and low FerrScore groups; Student’s t-test. **(I)** FerrScore of patients with different anti-PD-1 response; Wilcoxon test, *p < 0.05. **(J)** The ROC curve of FerrScore in predicting responsiveness of patients following anti-PD-1 therapy. **(K)** Kaplan–Meier survival analysis between high and low FerrScore groups in PRJEB23709 with anti-PD-1 + CTLA-4 combination therapy in melanoma. **(L)** Comparison of anti-PD-1 + CTLA-4 therapy response among high and low FerrScore groups; Student’s t-test. **(M)** Wilcoxon test of FerrScore variation in anti-PD-1 + CTLA-4 response. **(N)** The ROC analysis of FerrScore in predicting responsiveness of patients with anti-PD-1 + CTLA-4 therapy. **(O)** Kaplan–Meier survival analysis among high and low FerrScore groups of melanoma patients with adoptive T-cell therapy in GSE100797. **(P)** Variations of adoptive T-cell therapy within high and low FerrScore groups; Student’s t-test. **(Q)** FerrScore comparison in adoptive T-cell therapy; Wilcoxon test. ICI, immune checkpoint inhibitor; TMB, tumor mutational burden; TNB, tumor neoantigen burden; ROC, receiver operating characteristic; NS, no significance.

First, we investigated an anti-PD-L1 immunotherapy cohort (IMvigor210) of urothelial carcinoma. We divided patients into high and low FerrScore groups, and patients in the high FerrScore group had a worse OS (**Figure 7A**). Indeed, patients in the low FerrScore group were more likely to benefit from anti-PD-L1 therapy (**Figures 7B, C**). In addition, tumors with lower FerrScore also presented a higher frequency of tumor mutational burden (TMB) and tumor neoantigen burden (TNB) (**Figures 7D, E**). Higher TMB leads to higher TNB, increasing chances for T-cell recognition, and is clinically associated with better ICI therapy outcomes (33). The AUC value of the FerrScore model to predict anti-PD-L1 ICI benefits was 0.56 in the IMvigor210 cohort (**Figure 7F**). Second, we applied the FerrScore model to an anti-PD-1 therapy cohort (GSE78220) of melanomas, and patients with high FerrScore suffered worse OS (**Figure 7G**). Patients with low FerrScore had better anti-PD-L1 therapeutic response, and the AUC value of this model to predict anti-PD-L1 therapy response was 0.74 (**Figures 7H–J**). Third, we divided patients with melanoma (PRJEB23709) following anti-PD-1 + CTLA-4 therapy into high and low FerrScore groups. Patients with low FerrScore had better OS and benefited more from anti-PD-1 + CTLA-4 therapy (**Figures 7K–M**). The AUC value of the FerrScore model to predict anti-PD-1 + CTLA-4 therapy benefit was 0.61 (**Figure 7N**). Finally, we clustered patients with melanoma (GSE100797) following adoptive T-cell therapy into high and low FerrScore groups and found no difference in the OS and therapy benefits between the two groups (**Figures 7O–Q**). Collectively, the FerrScore was a promising biomarker to predict ICI therapy benefits to optimize therapy options for patients.

### SOCS1 and NOX4 are associated with immunity to TNBC

**Figure 8:**
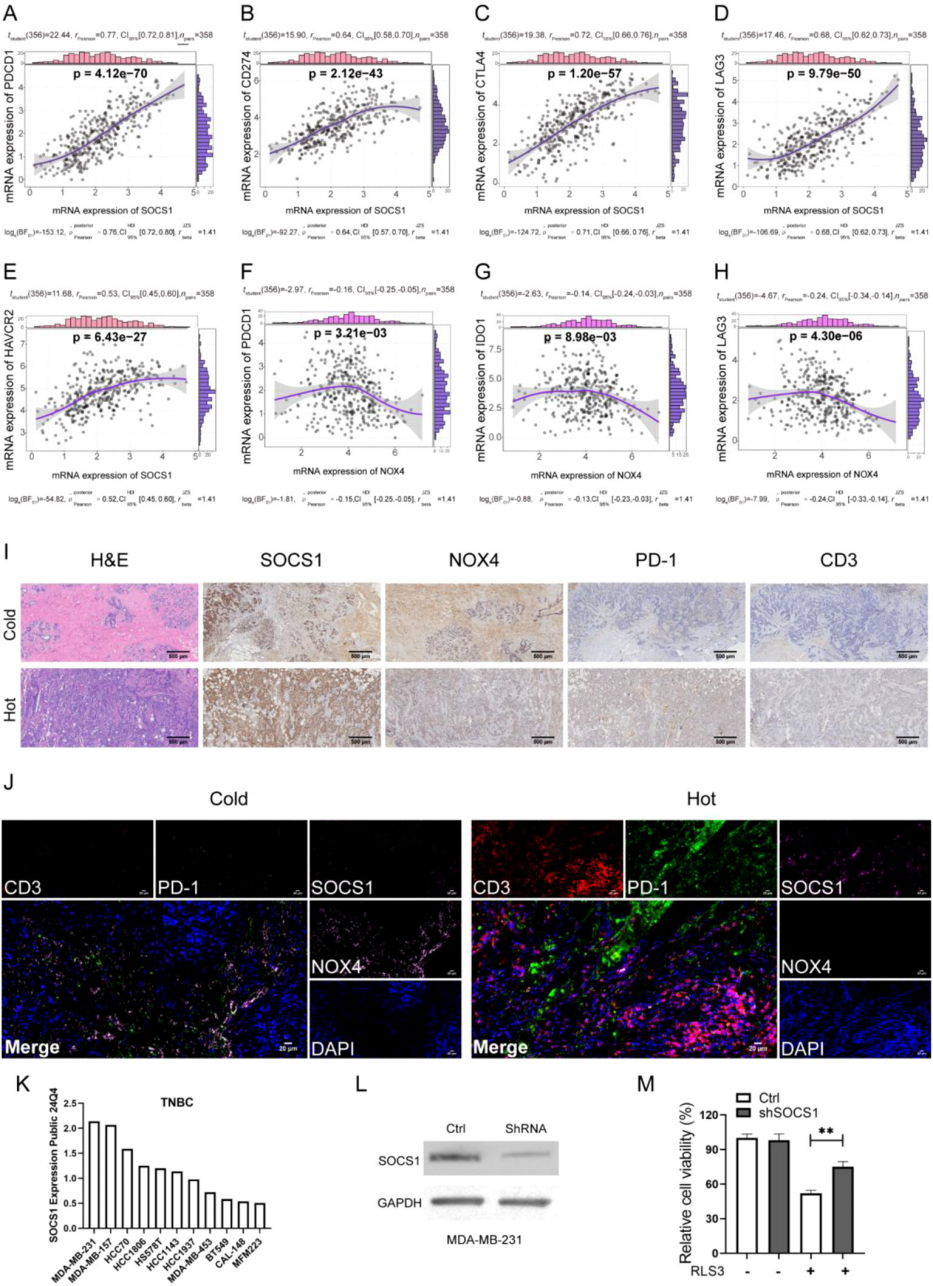
SOCS1 and NOX4 were correlated with the TME reshape in TNBC. **(A–E)** Pearson’s correlation analysis of the mRNA expression of SOCS1 and immune checkpoints (PD-1, PD-L1, CTLA-4, LAG3, and TIM3). **(F–H)** Pearson’s correlation analysis of the mRNA expression of NOX4 and immune checkpoints (PD-1, IDO1, and LAG3). **(I)** IHC detected the expression of SOCS1, NOX4, PD-1, and CD3 in two immune phenotypes of TNBC according to the spatial distribution of CD8^+^ T cells. Representative co-stained images are shown, and the scale bar corresponds to 500 μm. **(J)** The expression of SOCS1, NOX4, PD-1, and CD3 in two phenotypes of TNBC detected by four-color IF. Representative co-stained images are displayed, and the scale bar corresponds to 20 μm. **(K)** The expression levels of the SOCS1 gene across 11 TNBC cell lines using the DepMap portal (https://depmap.org/portal). **(L)** The knockdown efficiency of SOCS1 in MDA-MB-231 cells. **(M)** Cell viability in MDA-MB-231 cells (either control or shSOCS1) treated with RSL3 was assessed using the CCK-8 assay. **p < 0.01. TME, tumor microenvironment; TNBC, triple-negative breast cancer; IHC, immunohistochemical; IF, immunofluorescence.

We ultimately focused on SOCS1 and NOX4 relating to the previous analysis to further explore the definite role of FRG signature in TME reprogramming. Pearson’s correlation analysis indicated a strong positive correlation between SOCS1 expression and the mRNA expression of PDCD1 (PD-1), CD274 (PD-L1), CTLA-4, LAG-3, and HAVCR2 (TIM3), while the expression of NOX4 negatively correlated with PDCD1, IDO1, and LAG3 mRNA levels (**Figures 8A–H**). Therefore, we chose PD-1 as the key marker to study the relationship between the two FRGs and immune phenotype shaping. We divided the TNBC samples into cold and hot immune phenotypes according to CD3 IHC staining and examined the expression of SOCS1 and NOX4 across the two subtypes of tumors (**Figure 8I**). We found that tumors with high expression of NOX4 and low expression of SOCS1 were cold tumors, while tumors with high expression of SOCS1 as well as low expression of NOX4 exhibited phenotypes of hot tumors (**Figures 8I,J**). Here, we verified that SOCS1 and NOX4 actively participated in TME infiltration and remodeling of TNBC. Furthermore, we analyzed SOCS1 gene expression in 11 TNBC cell lines using the DepMap portal (https://depmap.org/portal). Notably, MDA-MB-231 cells exhibited the highest expression levels of SOCS1 (**Figure 8J**). Interestingly, the knockdown of SOCS1 expression in MDA-MB-231 cells led to a reduction in ferroptosis induced by RSL3 (**Figures 8L,M**).

## Discussion

Immunotherapy-activated CD8^+^ T cell-released IFNγ induces ferroptosis-specific lipid peroxidation in cancer cells to promote cancer immunotherapy (30). Ferroptotic tumor cell-derived DAMPs modulate the innate immune system and then reshape the TME (34). Ferroptosis in tumor cells has been proven to enhance response to radiotherapy, chemotherapy, and immunotherapy of tumors (16). In the present study, we identified eight survival-associated FRGs in the FUSCC cohort and explored the dysregulation and methylation of the eight FRGs in pan-cancers, as well as genetic variations and expression profiles in TNBC. We observed different imbalance of FRGs among pan-cancers, as well as expression profiles in TNBC, which resulted in the phenotypic heterogeneity of TNBC in clinical practice.

We classified patients with TNBC in the FUSCC cohort into two ferroptosis molecular subtypes with significantly different OS: cluster 1 (low FerrScore) and cluster 2 (high FerrScore). Tumors in cluster 2 had worse OS with lower immune, TIS, and estimate scores and higher TIDE scores, exclusion scores, and tumor purity, which indicate a negative correlation between ferroptosis and tumor immune infiltration. We also investigated a negative link between ferroptosis and immune-related pathways, such as natural killer cell-mediated cytotoxicity, and T-cell and B-cell receptor signaling pathways. Meanwhile, MHC complexes and immune checkpoints are rarely expressed in tumors of cluster 2, which may result from the almost absent infiltration of immune cells. Previous studies have implied that tumors with abundant lymphocyte infiltration exhibited better prognosis in a wide range of cancers (35). Additionally, there was a negative correlation between ferroptosis and the enrichment scores of the programmed cell death pathway, metal ion SLC transporters pathway, and drug metabolism cytochrome p450 pathway. To further explore the role of ferroptosis in tumor molecular subtyping and TME reprogramming, we constructed an RFG-based scoring system and defined it as FerrScore. We observed a negative correlation between FerrScore and the expression of MHC complexes and immune checkpoints. Tumors with high FerrScore had higher stromal, TIDE, and exclusion scores, as well as lower TIS and immune scores. Further, Pearson’s correlation analysis showed that tumors with higher FerrScore correlated with the significant enrichment of the metal ion SLC transporters pathway, tumor escape from immune attack pathway, and PD-1 signaling pathway. Our study indicated that ferroptosis in TNBC reprogrammed TME into a “cold” phenotype with less lymphocyte infiltration and immune checkpoint expression, which reduced the opportunities for T-cell recognition and reactivity to anticancer therapy.

Chemotherapy remains the preferred treatment option of advanced TNBC in clinical practice according to the 2022 version of the CSCO guidelines. However, resistance to chemotherapy remains a largely insurmountable challenge for cancer therapy. Ferroptosis inducers have been proven to act synergistically with traditional compounds (for example, cisplatin) to inhibit tumor growth in mouse models of head and neck tumor (32). We identified six CTRP-derived and six PRISM-derived drugs by Spearman’s correlation analysis for patients with high FerrScore, which provided a reference for clinical therapy options. We further confirmed everolimus as the most promising agent among 12 candidate drugs for patients with high FerrScore referring to comprehensive factors including CMap score, experimental evidence, and clinical trial status. Moreover, we applied FerrScore to four immunotherapy cohorts to predict ICI benefits. Our present study indicated that patients with lower FerrScore are more likely to benefit from anti-PD-L1, anti-PD-1, and anti-PD-1 + CTLA-4 ICI therapy. The PD-L1 inhibitor atezolizumab combined with nab-paclitaxel became the first immunotherapy regimen to show significant PFS and OS benefits in the first-line treatment of PD-L1-positive (≥1% tumor area) metastatic TNBC. Moreover, the US Food and Drug Administration (FDA) approved pembrolizumab plus chemotherapy for Metastatic Triple - Negative Breast Cancer (mTNBC) based on improved PFS in patients with a combined positive score (CPS) ≥10 (36). In summary, FerrScore was a potentially powerful metric to predict chemotherapy and ICI therapy benefits, which provided new perspectives for the clinical treatment of TNBC.

We focused on two ferroptosis genes, SOCS1 and NOX4, which showed a significant correlation with the expression of immune checkpoints of TNBC in the FUSCC cohort. Among the two immune phenotypes of TNBC according to CD3 expression, SOCS1 was significantly over-expressed in hot tumors, while NOX4 was highly upregulated in cold tumors of TNBC. Meanwhile, we investigated a positive correlation between the expression of SOCS1 and PD-1, while NOX4 exhibited a negative association with PD-1 expression by IHC and immunofluorescence (IF) staining. SOCS1, a negative feedback regulator of cytokine signaling, has been proven to suppress CD8^+^ T-cell proliferation in mouse models of acute inflammatory arthritis (37). SOCS1 promoted ferroptosis and correlated with clinical progression in TNBC (38, 39). SOCS1 was also identified as a negative regulator of PD-L1 by a barcoding system developed by Professor Brown (40). Meanwhile, SOCS1 was characterized as an intracellular negative checkpoint of CD4^+^ T cells, and inactivation of SOCS1 restored proliferation suppression and anticancer efficacy in both murine and human CD4^+^ T cells (41, 42). However, we found a positive correlation between SOCS1 expression and the infiltration of T cells CD4 memory activated and NK cells resting in TNBC, as well as PD-1 mRNA expression. Based on the above, we observed distinct roles and mechanisms of SOCS1 in PD-1 and PD-L1 expression in different disease types. Previous studies have shown that siRNA knockdown or pharmacologic inhibition of NOX4 promoted intra-tumoral CD8^+^ T-cell infiltration in many human cancer types and restored immunotherapy response induced by cancer-associated fibroblasts (CAFs) (43). NOX4 expression decreases from luminal to TNBC, with rising Reactive Oxygen Species (ROS) levels potentially driving mitochondrial reprogramming to promote aggressiveness (44). In summary, we verified the important influence of SOCS1 and NOX4 on immune landscape reprogramming and immune phenotype reshaping of TNBC by a variety of experiments, which implied their potential role in enhancing the immunotherapy effect of TNBC.

We still recognized some limitations of our research, although we applied the largest mRNA sequencing cohort of TNBC worldwide to identify ferroptosis-related molecular subtypes. First, TNBC is a heterogeneous disease characterized by its diverse molecular features, genetic variability, metabolic reprogramming, and unique tumor microenvironment. We focused exclusively on a Chinese cohort, which is not representative of the situation of all ethnographies, and patients from multicenter clinical queues should be included for further analysis and verification. Second, further mechanistic studies are urgently needed to explore how SOCS1 and NOX4 affect T-cell infiltration and PD-1 expression in TME of TNBC while some IHC and IF experiments have been conducted. Nevertheless, our group is conducting further studies that focus on the subject.

## Conclusion

In conclusion, we identified two distinct ferroptosis-related molecular subtypes with significantly different OS and immune landscapes, which illustrated a high heterogeneity of TNBC. We constructed a FerrScore based on eight FRGs and uncovered a negative correlation between FerrScore and immune infiltration, which reshaped the TME into an immunosuppressive phenotype. We confirmed everolimus as the most promising candidate agent for patients with high FerrScore, as well as documented FerrScore as a potentially powerful metric to predict anti-PD-L1, anti-PD-1, and anti-PD-1 + CTLA-4 ICI therapy benefits.

## Data Availability

The original contributions presented in the study are included in the article/**Supplementary Material**. Further inquiries can be directed to the corresponding authors.
