# Supplementary material for: Ferroptosis-related gene signature-based subtype identification of triple-negative breast cancer to prioritize treatment strategies
Source: Front Oncol. 2025 May 20;15:1541119. doi: 10.3389/fonc.2025.1541119 (PMC12129930; doi:10.3389/fonc.2025.1541119)
Supplement: Supplementary file 1 [file DataSheet1.docx]

**Supplementary figures**


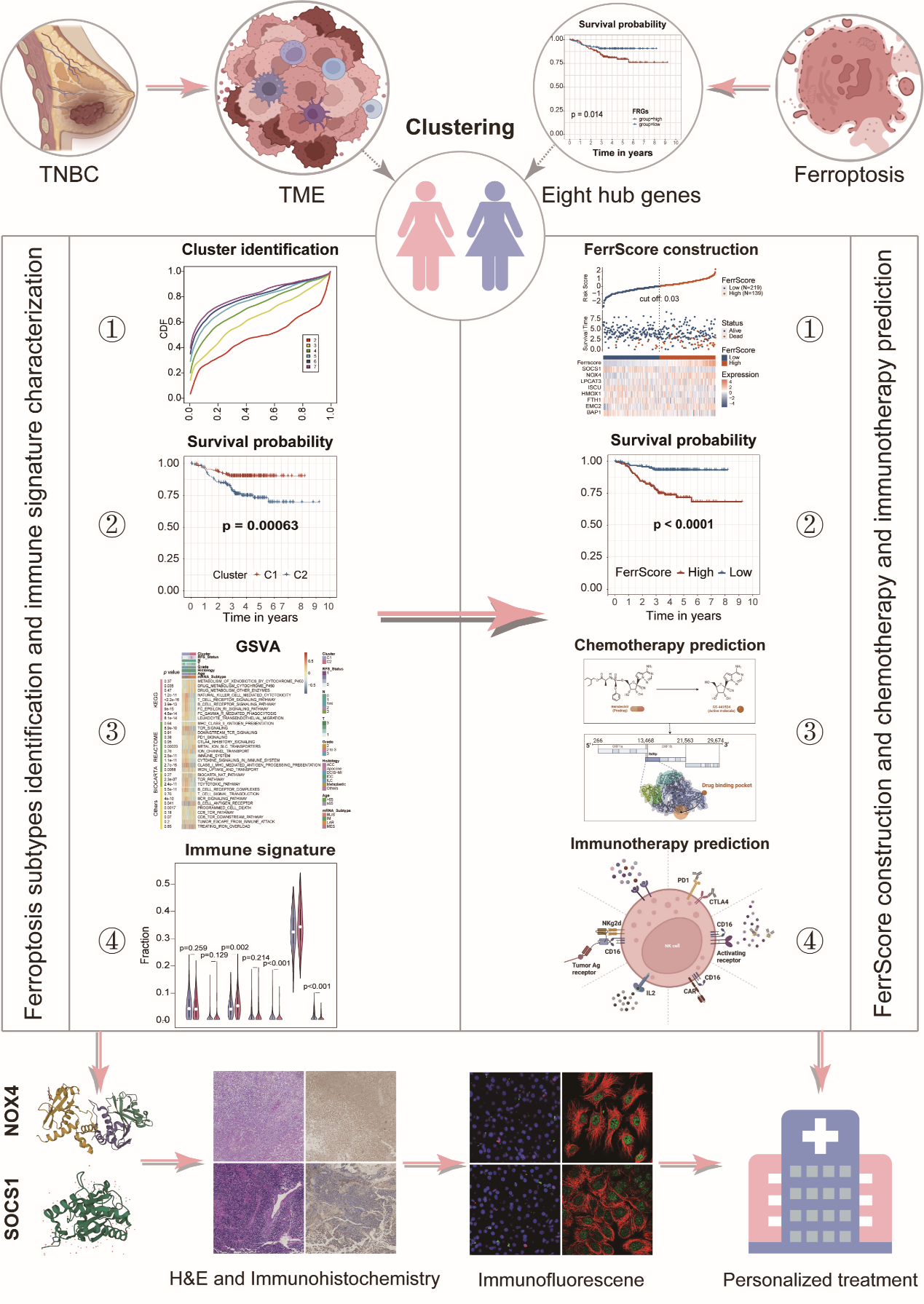


**Supplementary figure 1. Workflow chart of the present study.**


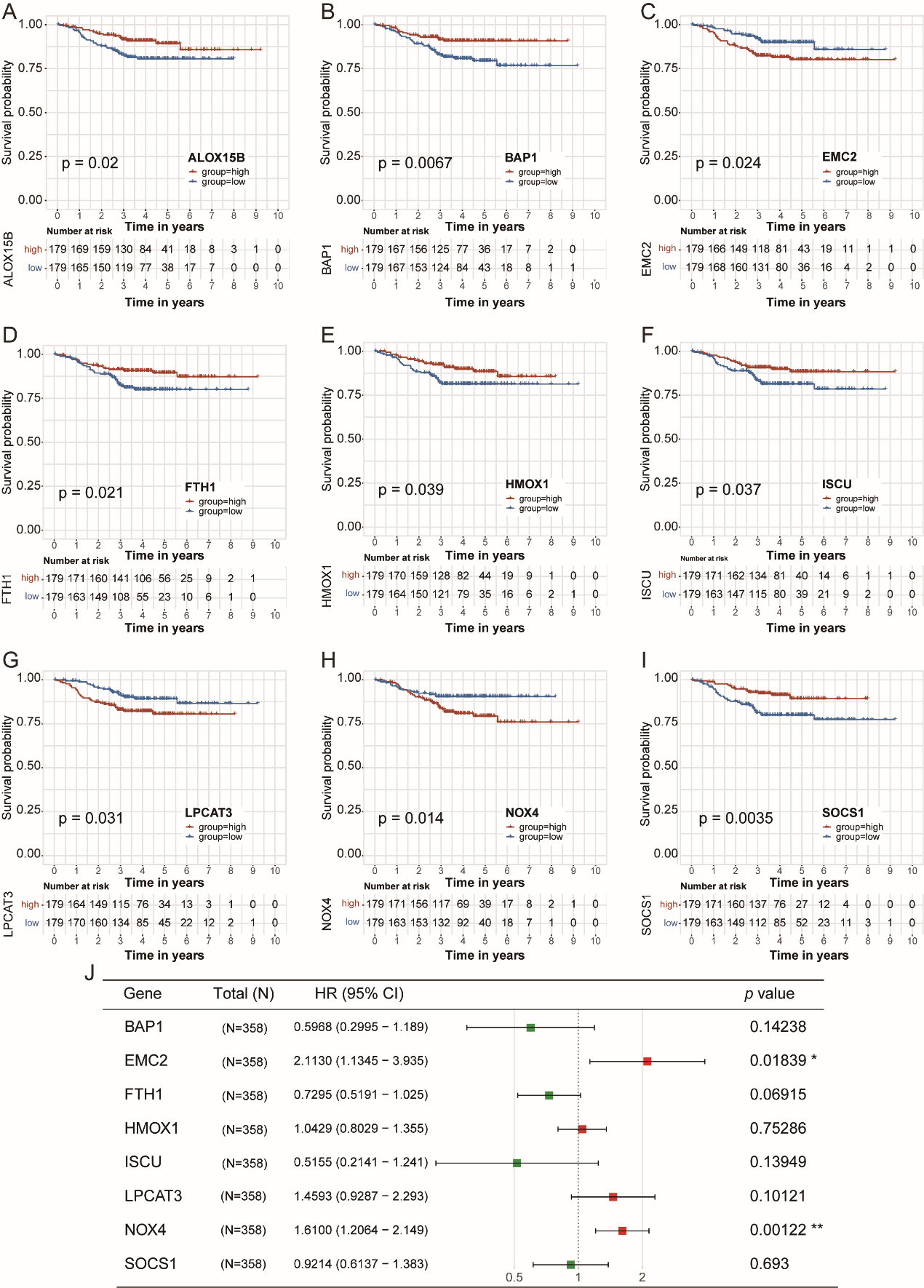


**Supplementary figure 2. Survival analysis of FRGs.** A-I. Kaplan-Meier survival analysis of FRGs in TNBC cohort. J. The forest plot showed the expression of the eight FRGs on OS of patients by univariate Cox regression analysis.

**
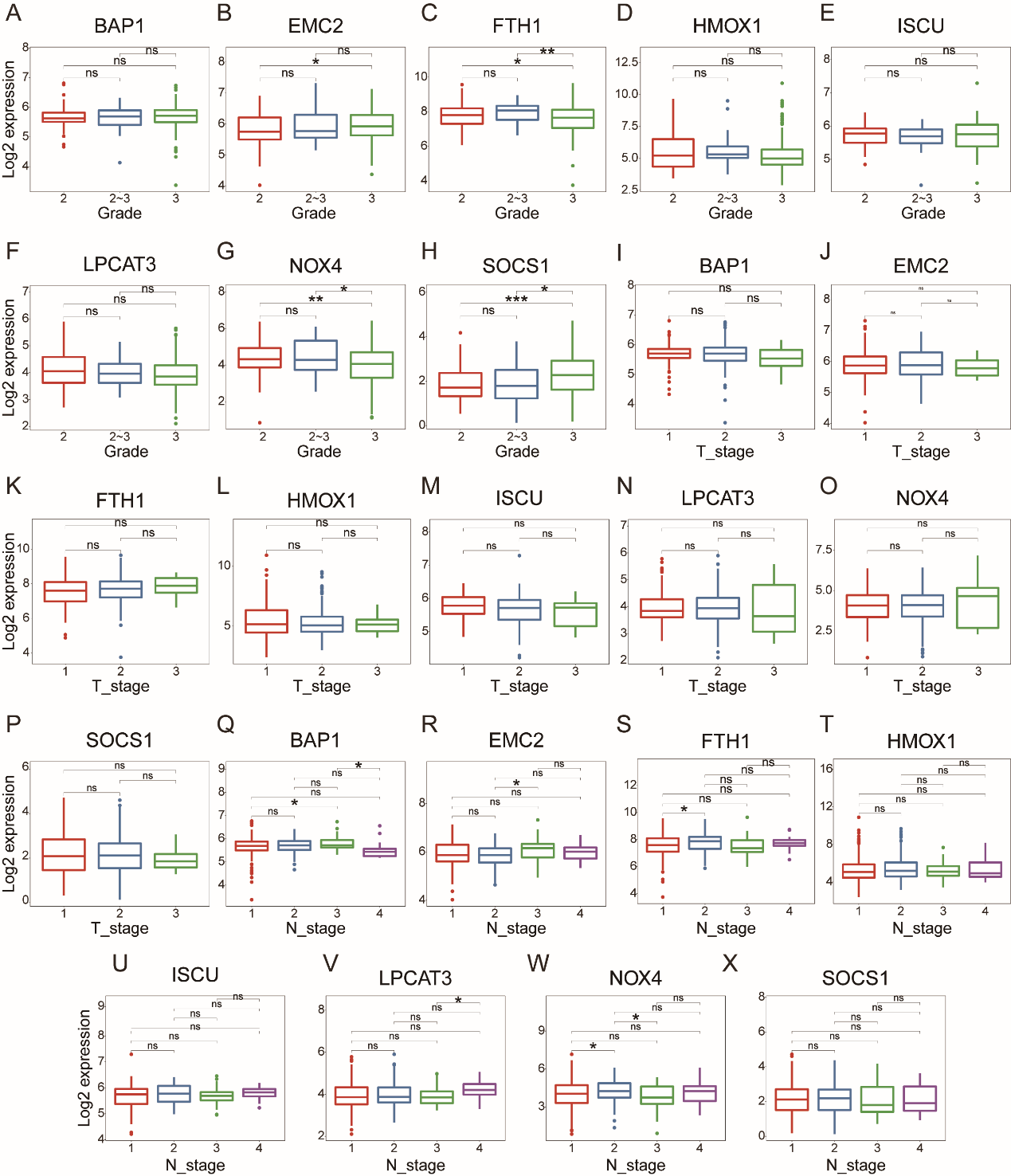
Supplementary figure 3. Expression variations of FRGs in pan-cancer.** A. The mRNA dysregulation of the eight FRGs in pan-cancer. B. Pearson correlation analysis between mRNA expression and CNV of the eight FRGs. C. Differential methylation of eight FRGs in pan-cancer. D. Pearson correlation analysis between mRNA expression and promoter methylation of eight FRGs in pan-cancer.

**
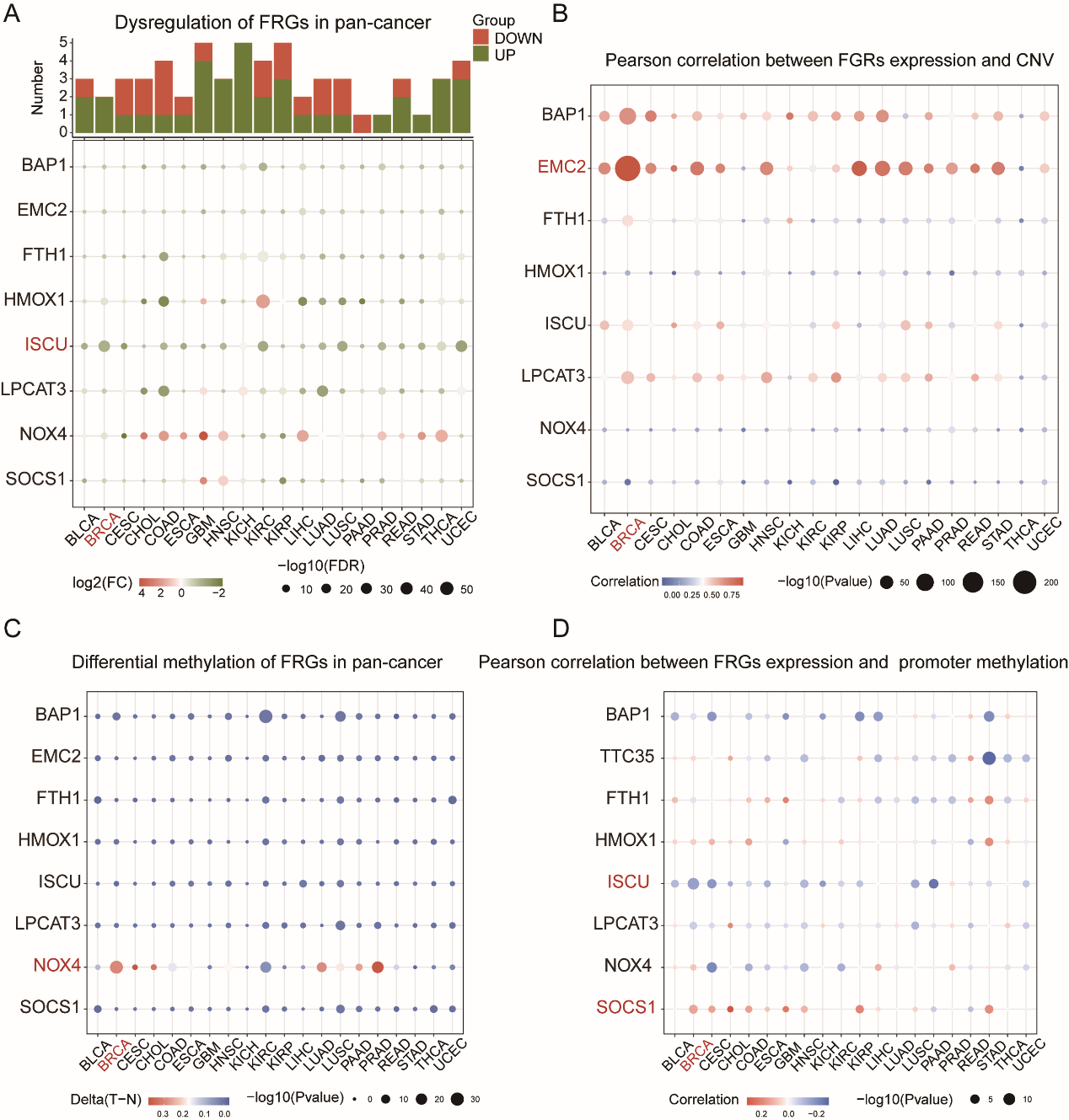
Supplementary figure 4. Expression profiles of FRGs in TNBC across different grades and stages.** A-H. Expression variations of FRGs among different grades of TNBC. I-P. Expression profiles of FRGs in 1-3 T stages of TNBC. Q-X. Comparison of mRNA expression between different N stages of TNBC; Student’s t-test, *p<0.05.

**
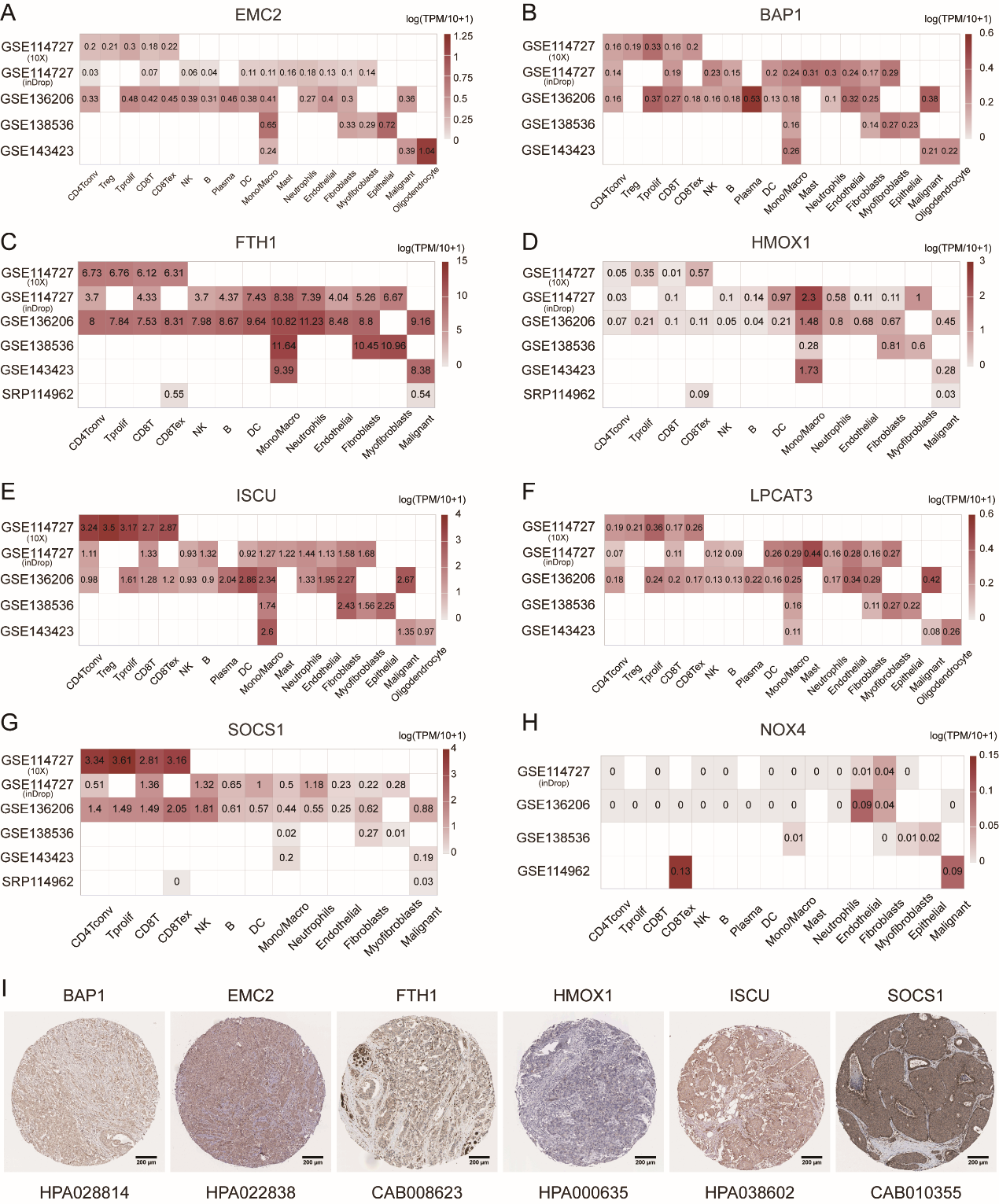
Supplementary figure 5. Expression of FRGs at the single cell and protein level.** A. Expression of the eight FRGs among malignant and immune cells across different scRNA-seq datasets. B. The spatial distribution of FRGs detected by IHC in breast cancer tissues from the Human Protein Atlas (HPA) (<https://www.proteinatlas.org/>).

**
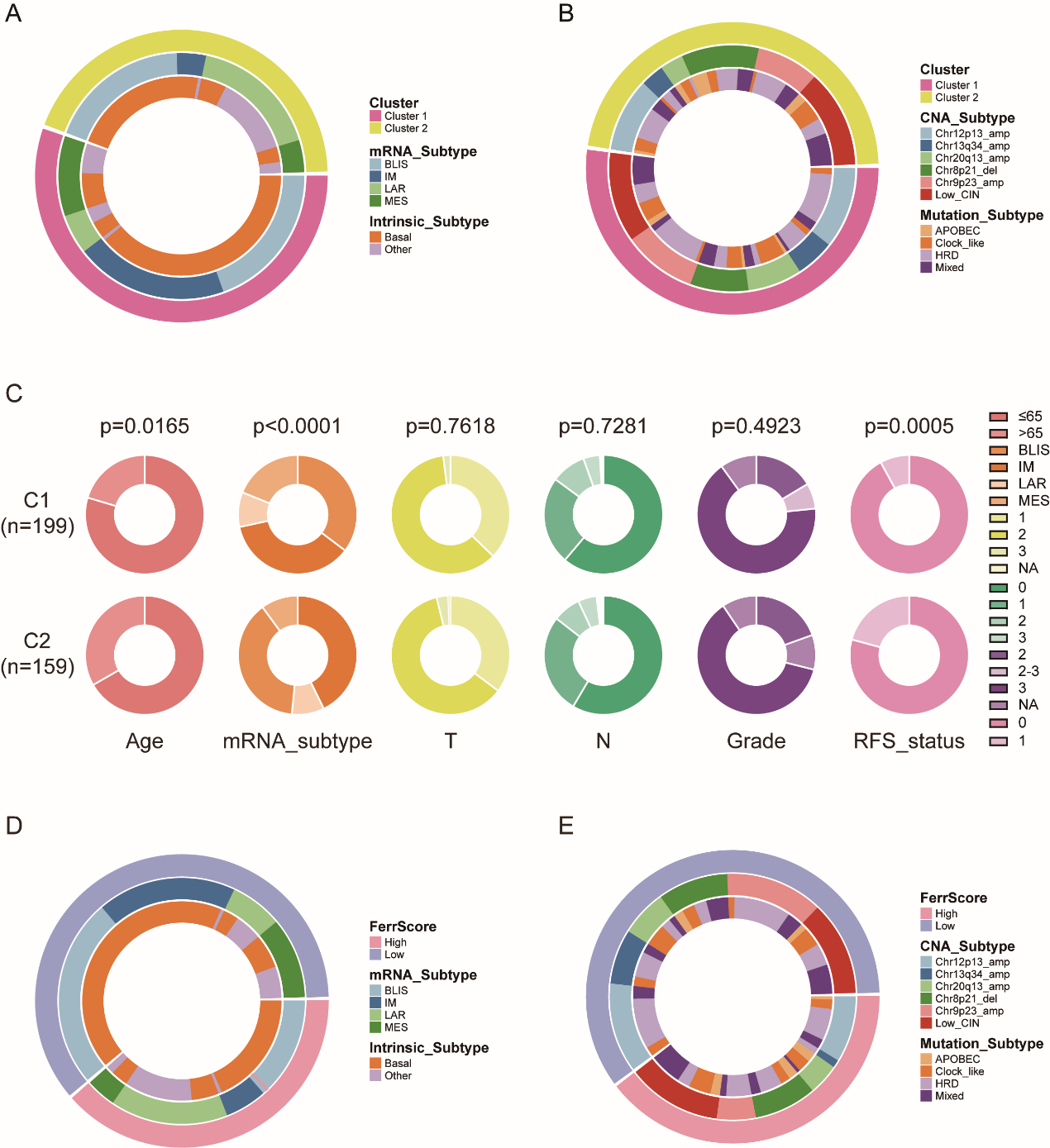
Supplementary figure 6. Correlation between ferroptosis clusters, FerrScore and Clinical significance in TNBC.** A-B. Correlation between clusters and mRNA subtype, intrinsic subtype, CNA subtype and mutation subtype. B. Variations of the clinical characteristics between two ferroptosis clusters. D-E. Correlation between FerrScore and mRNA subtype, intrinsic subtype, CNA subtype and mutation subtype.

**
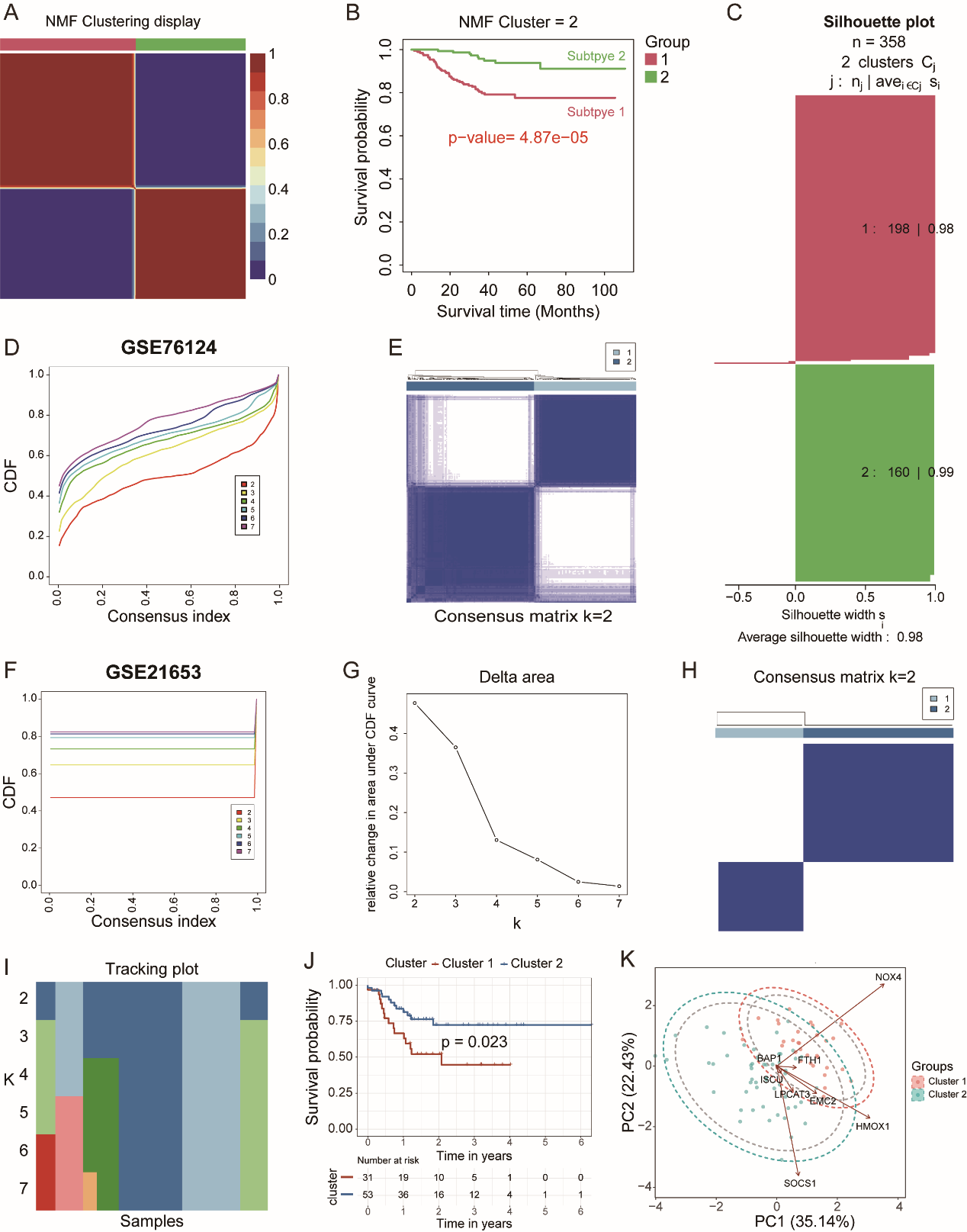
Supplementary figure 7. Validation of ferroptosis-stratified clusters in TNBC.** A. Ferroptosis subtypes clustering display using NMF algorithm by “CancerSubtypes” R package in FUSCC TNBC cohort. B. Kaplan-Meier survival analysis between the two ferroptosis clusters in FUSCC TNBC. C. The sihouette plot showed that the samples were split into two clusters of equal size. D-E. Unsupervised clustering identified two distinct clusters in GSE76124. F-I. Unsupervised clustering stratified patients into two ferroptosis subtypes in GSE21653 cohort. J. Kaplan-Meier survival analysis showed different survival probability between distinct ferroptosis subgroups. K. PCA revealed that ferroptosis clusters can be clearly distinguished by the expression of eight FRGs.

**
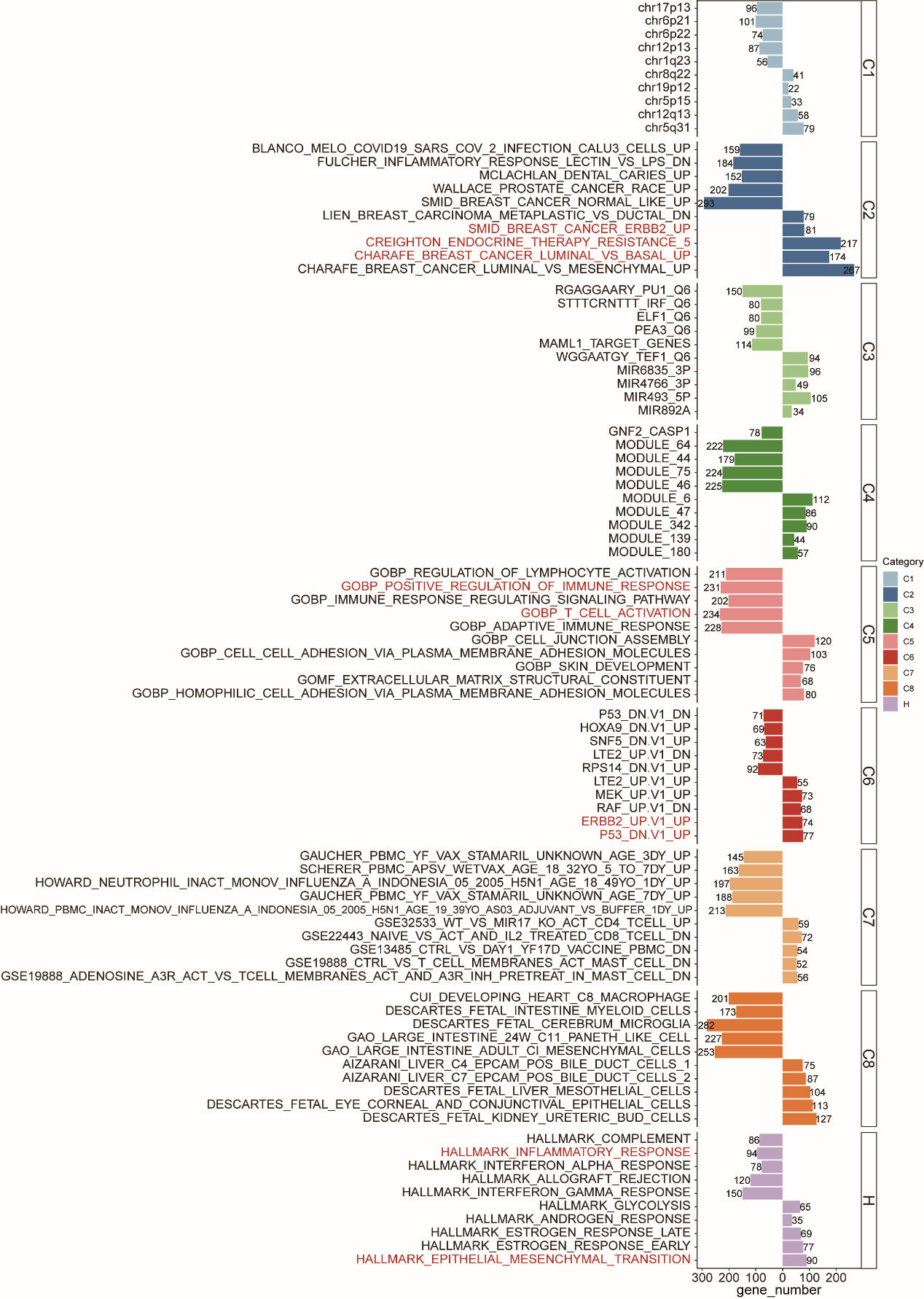
Supplementary figure 8. GSEA between the two ferroptosis subtypes of TNBC to identify significantly enriched pathways.**

**
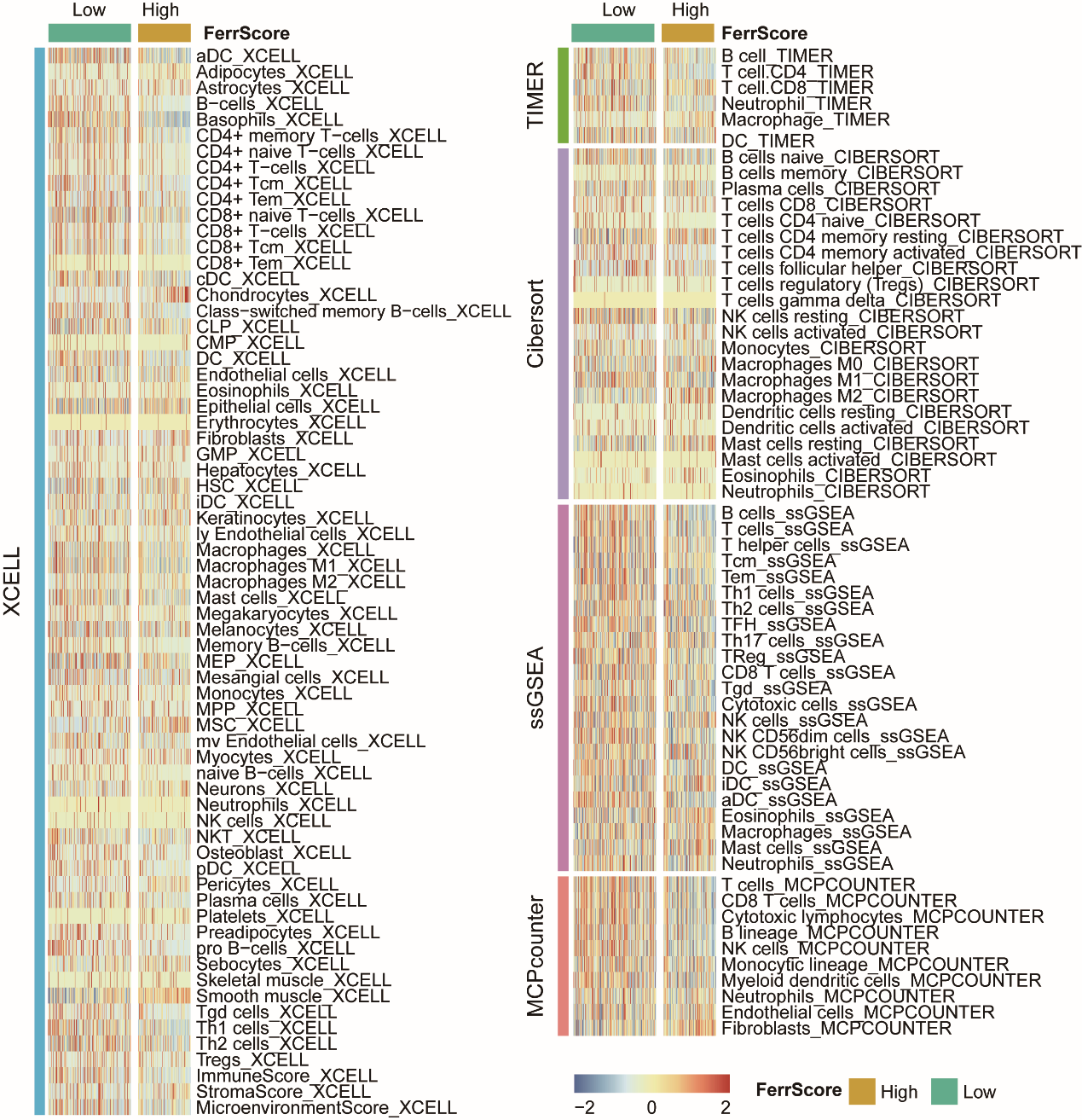
Supplementary figure 9. Correlation between FerrScore and immune infiltration of TNBC in FUSCC cohort.**

**
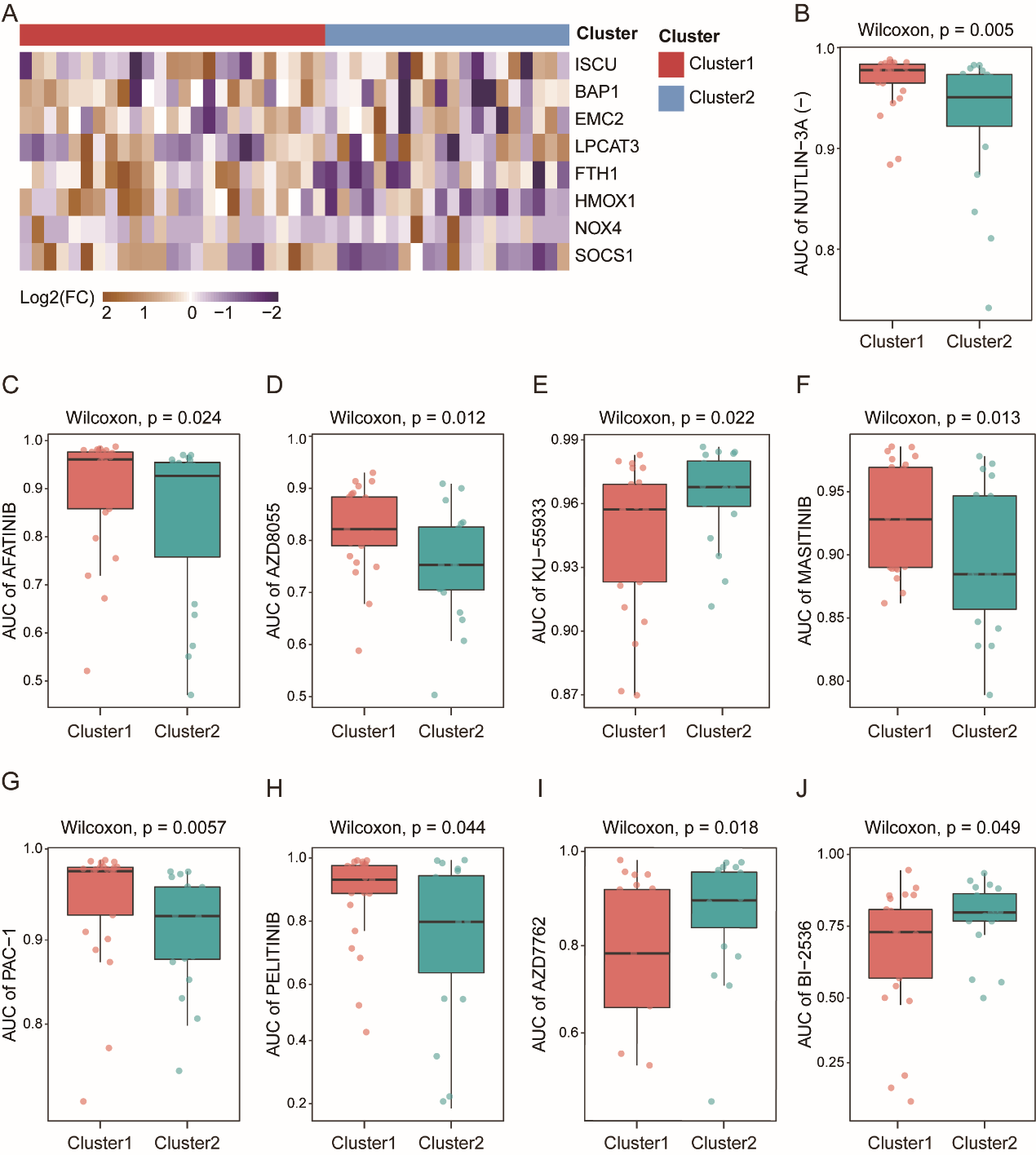
Supplementary figure 10. Candidate drug screening in FRGs-stratified clusters.** A. The heatmap showed the FRGs-stratified clusters in GDSC breast cancer cell lines. B-J. Comparison of the AUC value of NUTLIN-3A(-), AFATINIB, AZD8055, KU-55933, MASITINIB PAC-1, PELITNIB, AZD7762 and BI-2536 among two ferroptosis clusters.
